# A novel peptide derived from vascular endothelial growth factor prevents amyloid beta aggregation and toxicity

**DOI:** 10.1111/acel.13907

**Published:** 2023-07-06

**Authors:** P. Bouvet, P. de Gea, M. Aimard, N. Chounlamountri, J. Honnorat, J. G. Delcros, P. A. Salin, C. Meissirel

**Affiliations:** ^1^ MeLiS, Institut NeuroMyoGène (INMG), Synaptopathies and Autoantibodies, Institut National de la Santé et de la Recherche Médicale (INSERM), U1314 Centre National de la Recherche Scientifique (CNRS), UMR5284 Lyon France; ^2^ Univ Lyon Université Claude Bernard Lyon 1 Lyon France; ^3^ Centre de Recherche en Cancérologie de Lyon, Apoptosis, Cancer and Development, Institut PLAsCAN, INSERM U1052, CNRS UMR5286 Centre Léon Bérard Lyon France; ^4^ Centre de Recherche en Cancérologie de Lyon, Small Molecules for Biological Targets INSERM U1052 – CNRS UMR5286, ISPB Rockefeller Lyon France; ^5^ Centre de Recherche en Neurosciences de Lyon, Forgetting Processes and Cortical Dynamics INSERM U1028, CNRS UMR5292 Bron France

**Keywords:** Alzheimer's disease, amyloid beta‐peptides, blocking peptide, synapses, synaptic transmission, VEGF

## Abstract

Amyloid‐β oligomers (Aβo) are the most pathologically relevant Aβ species in Alzheimer's disease (AD), because they induce early synaptic dysfunction that leads to learning and memory impairments. In contrast, increasing VEGF (Vascular Endothelial Growth Factor) brain levels have been shown to improve learning and memory processes, and to alleviate Aβ‐mediated synapse dysfunction. Here, we designed a new peptide, the blocking peptide (BP), which is derived from an Aβo‐targeted domain of the VEGF protein, and investigated its effect on Aβ‐associated toxicity. Using a combination of biochemical, 3D and ultrastructural imaging, and electrophysiological approaches, we demonstrated that BP strongly interacts with Aβo and blocks Aβ fibrillar aggregation process, leading to the formation of Aβ amorphous aggregates. BP further impedes the formation of structured Aβo and prevents their pathogenic binding to synapses. Importantly, acute BP treatment successfully rescues long‐term potentiation (LTP) in the APP/PS1 mouse model of AD, at an age when LTP is highly impaired in hippocampal slices. Moreover, BP is also able to block the interaction between Aβo and VEGF, which suggests a dual mechanism aimed at both trapping Aβo and releasing VEGF to alleviate Aβo‐induced synaptic damage. Our findings provide evidence for a neutralizing effect of the BP on Aβ aggregation process and pathogenic action, highlighting a potential new therapeutic strategy.

## Graphical abstract

We designed a peptide derived from VEGF that has the ability to interfere with Aβ aggregation, blocking fibril formation. This Blocking Peptide further impedes the production of Aβ oligomers and counteracts their synaptic targeting. Remarkably, acute treatments with the Blocking Peptide efficiently rescue the severe deficits in hippocampal synaptic plasticity in the APP/PS1 mouse model of AD.

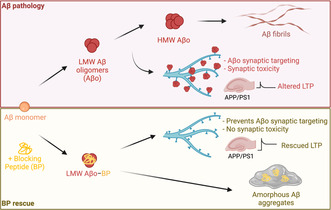

**Abbreviations**
AD: Alzheimer's disease
Aβ: Amyloid‐βpeptide
Aβm: Amyloid‐β monomer
Aβo: Amyloid‐β oligomer
APP/PS1: Amyloid precursor protein/presenilin‐1 transgenic mouse
BP: Blocking peptide
CP: Control peptide
fEPSPs: Field excitatory postsynaptic potentials
HMW: High‐molecular weight
KO: Knockout
LMW: Low‐molecular weight
LTP: Long‐term potentiation
NFT: Neurofibrillary tangles
PPF: Paired‐pulse facilitation
PS1: Presenilin 1
SEM: Standard error of the mean
TBS: Theta burst stimulation
TEM: Transmission electron microscopy
ThT: Thioflavin‐T
VEGF: Vascular endothelial growth factor
VEGFR2: Vascular endothelial growth factor receptor 2
WT: Wild type

## 1 INTRODUCTION

Alzheimer's disease (AD), the most common neurodegenerative disease, is initially characterized by a progressive memory loss and decline in cognitive abilities which leads to dementia (The Alzheimer's Association, 2021). The neuropathological process begins decades before the symptomatic phase of the disease and involves the accumulation of insoluble protein aggregates in the brain. These aggregates have been characterized as the two main histopathological markers of the disease, the neurofibrillary tangles (NFT) due to an hyperphosphorylation of the Tau protein within neurons and the amyloid β plaques caused by the accumulation of the β‐amyloid peptide (Aβ) in the extracellular space (Long & Holtzman, 2019). Because familial forms of AD are due to gene mutations of proteins involved in Aβ production, Aβ plaques were first proposed to play a causal role in AD pathogenesis, leading to the amyloid hypothesis (Hardy & Higgins, 1992). However, Aβ plaque formation in AD patient's brain does not correlate with cognitive symptoms, in contrast to NFT (Braak & Braak, 1991) and to brain levels of soluble Aβ forms (Lambert et al., 1998; Lue et al., 1999; McLean et al., 1999), revisiting the original amyloid hypothesis (Benilova et al., 2012).

Strong evidence now indicates that soluble Aβ oligomers (Aβo) produced by the self‐aggregation of the two most common Aβ_40_ and Aβ_42_ isoforms of the Aβ peptide are the most toxic Aβ species (Mucke & Selkoe, 2012). Indeed, Aβo derived from AD patients' brain, cell lines overexpressing human Aβ or synthetic preparations have been shown to induce synaptotoxicity (Ferreira et al., 2015) and to alter hippocampal long‐term potentiation (LTP), a cellular basis of learning and memory (Selkoe, 2008; Shankar et al., 2008; Walsh et al., 2002). Thus, Aβ aggregation process is considered as a primary cause in AD pathogenesis. However, Aβo are transient intermediates with a dynamic and heterogeneous nature and major efforts have been produced to identify the bioactive species responsible for neurotoxicity in AD. Notably, Aβo have been classified by size, shape, structure, or by their reactivity to conformation‐dependent antibodies (Ashe, 2020; Chen et al., 2017). High molecular weight (HMW) Aβo over 50 kDa have been shown to target synapses more efficiently than low molecular weight (LMW) Aβo (Lacor et al., 2004, 2007) and to be the predominant soluble Aβ species in AD brain (Yang et al., 2017). Although HMW Aβo are considered less toxic for synapses than smaller species into which they can dissociate, such as dimers (Shankar et al., 2008; Yang et al., 2017), they have been shown to cause neurotoxicity and synapse dysfunction in hippocampal slices (Yasumoto et al., 2019). Thus, preventing synapse failure by targeting Aβo toxic species and increasing synaptic protection could be a beneficial strategy to prevent cognitive decline in AD.

The Vascular endothelial growth factor (VEGF) and its main receptor VEGFR2 are now considered key players in hippocampal synaptic plasticity and memory. Thus, increasing brain VEGF levels in animal models improves memory performances, while invalidating VEGFR2 in neurons impairs them (Cao et al., 2004; De Rossi et al., 2016; Licht et al., 2011). In humans, higher VEGF levels in cerebrospinal fluid have been associated with less hippocampal volume changes and cognitive decline overtime in healthy and AD patients (Hohman et al., 2015). This potential neuroprotective role of VEGF has been confirmed in AD mouse models, where brain injections of VEGF‐producing nanoparticles, microcapsules, or mesenchymal stem cells reduced brain Aβ load and learning and memory impairments (Garcia et al., 2014; Herrán et al., 2013; Spuch et al., 2010). Recently, we investigated the cellular mechanisms involved in the protective role of VEGF and demonstrated that it counteracts Aβo toxic action by preventing LTP impairment and synapse morphological and functional changes. However, this beneficial effect of VEGF might be limited in AD because we also uncovered that Aβo directly interacts with VEGF (Martin et al., 2021). Taking advantage of this interaction, we designed a new peptide tool, named the blocking peptide (BP), mimicking part of the VEGF linear protein sequence which is specifically recognized by Aβo.

Here, we propose to use BP to specifically target Aβo and prevent their synaptotoxicity. We showed that BP is able to bind Aβo with high affinity and to block the process of Aβ fibrillar aggregation. Moreover, we revealed that BP impedes the formation of structured Aβo which are specifically recognized by a conformational antibody known to selectively target Aβo toxic for synapses. Consistent with this finding, BP successfully inhibits Aβo binding to synapses in hippocampal neurons. Importantly, we uncovered that acute BP treatment can rapidly restore LTP in slices from aged APP/PS1 mice. We finally revealed that BP prevents Aβo‐VEGF interaction, potentially releasing VEGF and restoring its protective effect on synapses and LTP.

## 2 MATERIALS AND METHODS

### 2.1 Animals

Animal specimens Embryonic day 17–18 (E17–18) C57Bl/6JRj wild‐type male and female mice were used for primary hippocampal cell cultures (Table S1). Electrophysiological field potential recording experiments and brain section immunostainings were performed on 8‐month‐old wild type and transgenic heterozygous *APP/PS1‐21* mice generated on a C57Bl/6 background and expressing a transgene combining human *APP* with the Swedish mutation (*APP* ^KM670/671NL^) and mutated *L166P* human presenilin 1 (*PS1*) under the *Thy1* promoter (Radde et al., 2006). Only male mice were used. Genotyping was carried out to reveal the presence or absence of *APP* and *PS1* transgenes. The study was conducted in accordance with the European Community Council directive 2010/63/EU on the protection of animals used for experimental and scientific purposes. Animal care and treatment procedures were realized according to the guidelines approved by the French Ethical Committee of the Lyon 1 University (DR2013‐47).

### 2.2 Aβ preparation and aggregation

Synthetic Aβ_40_, Aβ_42_, and their biotinylated forms were obtained as lyophilized samples from Bachem (Aβ_40_, b‐Aβ_40_, Aβ_42_ and b‐Aβ_42_). Briefly, peptides were solubilized in 1,1,1,3,3,3‐hexafluoro‐2‐propanol to prevent oligomerization, then evaporated overnight under a chemical fume hood, and stored as a dried peptide film at −80°C until use, as previously described (Stine et al., 2003). Aβ monomers (Aβm) were prepared extemporaneously by first dissolving the peptide film in 2 mM dimethyl sulfoxide (DMSO) and vortexing for 1 min. Peptides were further diluted to 100 μM in ice‐cold PBS. Diluted peptides were subsequently sonicated for 20 min at 4°C, centrifuged at 10,000*g* for 3 min, then the supernatant was collected and concentration of Aβm measured using a micro BCA protein assay kit, prior to being aliquoted and stored at −20°C until use. Typically, this centrifugation step resulted in the loss of around 50%–60% of the initial Aβ amount. Aβ preparations were used either directly as monomers (for Aβ_40_ Elisa assays and ThT experiments) or were subjected to different oligomerization states, enriched in high molecular weight Aβ_42_ oligomers (HMW Aβo) or in low molecular weight Aβ_42_ oligomers (LMW Aβo). For Aβ preparations enriched in Aβ fibrils, monomeric Aβ_42_ peptides (Aβm) were incubated at a concentration of either 30 μM or 15 μM during 24 h at 37°C. For HMW Aβo, Aβm was incubated at 15 μM for 2 h at 37°C. For ELISA assays, we also used a LMW Aβo preparation (Stine et al., 2003) by incubating biotinylated Aβm at 4°C for 24 h. LMW Aβo were then centrifuged at 10,000 *g* for 10 min and the supernatant was collected.

In all the experiments Aβo concentrations are expressed as monomer equivalent concentrations, as previously reported (Laurén et al., 2009).

### 2.3 Blocking and control peptides preparation

Blocking peptide (BP) (seq: KRKKSRYKSWSVYVG) and control peptide (CP) (seq: NDYKDDDDKGAAA) were obtained as lyophilized samples from Proteomic Solutions and stored at −20°C until reconstitution. The sequence design comes from previous peptide array experiments aimed at studying Aβo‐VEGF interaction (Martin et al., 2021). Based on the level of hydrophobicity and positive/negative charges, BP stock solution was prepared at 20 mM in 10% acetic acid and CP stock solution at 20 mM in 50% DMSO and 5% ammonium bicarbonate. Then, the peptides were aliquoted and stored at −80°C until use.

### 2.4 ELISA assays

The binding of CP or BP to synthetic biotinylated LMW Aβo (oligomerized at 4°C for 24 h) or Aβ40 monomers (Aβm) was determined by indirect ELISA in which CP or BP were used as the capture antigens and the biotin tag of the biotinylated Aβ peptides for the detection. 96‐well clear polystyrene microplate were coated overnight at 4°C with 5 μM of CP or BP in PBS, pH 7.4. After immobilization, the plate was washed three times with PBS 0.05% Tween 20, and various concentrations of biotinylated Aβ peptides were added in triplicate from 0.1 μg/mL to 10 μg/mL (21 nM to 2.1 μM for LMW Aβo, 22 nM to 2.2 μM for Aβm) and incubated for 2 h at RT. After additional washes with PBS 0.05% Tween 20, HRP‐conjugated streptavidin (1/40) was added for 20 min at RT, followed by a washing step and an incubation in substrate reagent containing 3,3′,5,5′‐ Tetramethylbenzidine (TMB) and H_2_O_2_. The reaction was stopped with a solution containing H_2_SO_4_. Absorbance was successively measured at 450 and 540 nm with a TECAN microplate reader and optical density values at 540 nm were subtracted from the ones at 450 nm to correct for optical imperfections of the plate. Unspecific Aβ binding to the microplate was determined using non‐coated wells.

### 2.5 Electrophysiology

Local field potential recordings (LFP) were performed on acute coronal hippocampal slices from 8‐month‐old wild type and transgenic heterozygous male APP/PS1‐21 mice to measure baseline synaptic response and long‐term potentiation (LTP) of Schaffer collaterals to CA1 (SC‐CA1) pyramidal cell synapses. Hippocampal slices (400 μm thick) were cut using a vibratome (Leica VT1200S) and incubated at room temperature in artificial cerebrospinal fluid (ACSF) containing (in mM): 124 NaCl, 10 glucose, 1.25 NaH_2_PO_4_, 2.5 KCl, 26 NaHCO_3_, 1.3 MgCl_2_, and 2.5 CaCl_2_, bubbled with 95% O_2_ and 5% CO_2_, pH 7.4 for 1 h prior to recording. Field excitatory postsynaptic potentials (fEPSPs) were recorded extracellularly in stratum radiatum of CA1 region of the dorsal hippocampus in presence of gamma‐aminobutyric acid (GABA)‐A receptor antagonist picrotoxin (100 μM). Electrical stimulation was realized with a bipolar tungsten electrode placed in CA1 stratum radiatum and fEPSPs were measured using borosilicate glass microelectrodes (~1–3 MΩ) filled with ACSF. LFP were amplified and low‐pass filtered at 3 kHz using a differential amplifier (×1000, WPI) and data acquisition and analyses were carried out using a National Instrument interface coupled with Elphy software (G. Sadoc, CNRS, France). LFP were sampled at 10 kHz and fEPSPs initial slope was computed to quantify synaptic responses. Paired‐pulse stimulation of evoked responses at SC‐CA1 synapses was carried out using a 50 ms inter‐stimulus interval. Paired‐pulse stimulation of synaptic responses at short intervals evoked paired‐pulse facilitation (PPF) of responses at the SC‐CA1 synapses. PPF represents a form of short‐term plasticity and the modulation of the paired‐pulse ratio (PPR) of evoked synaptic responses has been shown to depend on presynaptic mechanisms of neurotransmitter release (i.e., modulation of the glutamate release probability at SC‐CA1 synapses). For quantification, the slope of the synaptic response to the second stimulation was divided by that of the first response.

After baseline recordings of synaptic activity evoked at 0.2 Hz for at least 10 min, slices were incubated in ACSF or treated with CP or BP at 0.5 μM for 40 min before inducing LTP with the theta burst stimulation (TBS). The effects of the peptides on synaptic responses were tested in a series of pilot experiments on mice of both genotypes. In contrast to the application of 2 μM of BP, a 30 min administration of 0.5 μM had no effect on synaptic transmission (Figure S3A,B). The 0.5 μM concentration of BP did not induce a change in these responses during the 40 min prior to LTP induction at the SC‐CA1 synapses. TBS consisted of 10 trains separated by 30 s, each train composed of 10 bursts at 5 Hz and each burst providing 4 pulses at 100 Hz. Before LTP induction, precautions were taken to ensure that approximately the same amplitude of fEPSPs was obtained in baseline for the different experiments to achieve the same level of cooperativity in each group. After LTP induction, fEPSPs were recorded for at least 60 min.

### 2.6 Cell cultures and treatments

#### 2.6.1 Hippocampal neurons

Primary hippocampal neuron cultures were prepared from E17‐18 C57Bl/6JRj mice. Briefly, hippocampi were removed and collected in Hank's buffered salt solution (HBSS), digested in HBSS supplemented with trypsin (0.25% v/v) for 10 min at 37°C and then rinsed in HBSS and BSA 0.2%. Next, hippocampi were triturated in Neurobasal medium without phenol red, supplemented with L‐glutamine (2 mM), 2% B27, 1% penicillin/streptomycin (P/S) (10,000 U/mL). Hippocampal neurons were then plated onto poly‐l‐lysine coated coverslips (0.5 mg/mL) at a low density (15 × 10^3^ cells/cm^2^) for synaptic targeting experiments. Neurons were cultured in supplemented Neurobasal medium at 37°C under 5% CO_2_, one‐half of the medium being changed once a week.

For synaptic targeting experiments, hippocampal neuron cultures were used after 21 days in vitro (DIV) and treated for 30 min with a final concentration of 0.5 μM biotinylated Aβ preparations enriched in HMW Aβo. HMW Aβo enriched preparations were derived from Aβm which have been incubated at 15 μM in the presence or not of equimolar concentration of CP or BP, for 2 h at 37°C.

### 2.7 Western and dot blotting

#### 2.7.1 Western blotting

Aβm was aggregated at 15 μM with or without CP or BP at equimolar concentrations, during 2 h at 37°C (enriched in HMW Aβo) or 24 h at 37°C (enriched in Aβ fibrils). After a 5 min denaturation step at 95°C in Laemmli/DTT (1,4‐Dithiothreitol), Aβ preparations (1 μg) were subjected to immunoblotting analysis. Aβ assemblies were separated on 4–12% gradient SDS‐PAGE gels at 120 V, with cathode and anode containing MES buffer. Gels were then transferred on 0.2 μm nitrocellulose membranes at 120 V for 1 h in 20% methanol, Tris‐Glycine buffer.

After a 45 min blocking step in Tris‐buffered saline, 0.1% Tween, pH 7.6, 5% milk, membranes were immunoblotted overnight at 4°C with dedicated antibodies (Table S1) diluted in Tris‐buffered saline, 0.1% Tween, pH 7.6, with 2% milk. Appropriate horseradish peroxidase (HRP)‐conjugated secondary antibodies were next applied for 1 h30 at RT. Proteins were visualized with an ECL detection system and band intensities quantified using a densitometric analysis with ImageJ software.

#### 2.7.2 Dot blotting

For dot blots, each sample including Aβm, Aβo, or Aβ aggregated from 0 to 2 h with or without BP at 15 μM, was adsorbed onto a 0.2 μm nitrocellulose membrane, blocked with 5% milk in TBS without detergent at RT for 45 min, and incubated overnight at 4°C with the anti‐Aβ oligomer antibody A11 (kindly provided by Rakez Kayed, University of Texas, USA) in TBS with 2% milk. Following washes, a goat anti‐rabbit secondary antibody conjugated to HRP was incubated for 1 h30 at RT before detection by chemiluminescence using SuperSignalTM West Pico chemiluminescent Substrate kit and ChemiDoc imaging system. For epitope mapping experiments, Aβ samples were adsorbed prior to an incubation with the N‐terminal anti‐ Aβ_1–16_ (6E10) and/or anti‐Aβ_17–24_ (4G8) or anti‐Aβ_22–35_ antibody for 2 h at RT in TBS‐Tween with 2% milk. After washes, biotinylated BP at 5 μM was incubated or not overnight at 4°C in TBS‐Tween followed by additional washes and an incubation with an anti‐biotin secondary antibody conjugated to HRP for 1 h30 at RT. Bound biotinylated BP was visualized by chemiluminescence. In some experiments, the anti‐Aβ_22–35_ was applied overnight after the BP followed by an anti‐rabbit secondary antibody conjugated to HRP to detect Aβ unbound to BP. Quantification of signal intensities was performed using a densitometric analysis with the ImageJ software.

### 2.8 Thioflavin T assays

Thioflavin‐T (ThT) assays were performed in black polystyrene 384‐well plates using a Tecan microplate reader with a set of excitation/emission wavelengths of 450/490 nm. Monomeric Aβ_42_ was prepared as described above and CP or BP were used at indicated concentrations, ranging from 0.5 to 15 μM. The final concentration of ThT was 20 μM, and Aβm was used at 15 μM in PBS buffer (pH 7.4), with a total reaction volume per well of 30 μL. The plate was sealed with a transparent plastic film and incubated at 37°C prior to fluorescence measurement. ThT fluorescence intensity was monitored every 5 min over 24 h at 37°C without agitation. Three replicates per condition were measured.

### 2.9 Hippocampal neuron immunostaining

Treated hippocampal neuron cultures were fixed for 4 min in 4% paraformaldehyde (PFA) in 0.1 M phosphate buffer at RT, prior to immunostaining. After a blocking step in non‐permeabilizing condition (PBS, 1% BSA) surface‐bound biotinylated Aβ was detected with fluorescently tagged streptavidin incubated for 3 h at RT. Three washes in PBS were then performed followed by a permeabilizing step in PBS, 0.3% Triton‐X‐100 and 1% BSA for 1 h, prior to an overnight incubation at 4°C with antibodies directed against PSD95 and Bassoon in the permeabilizing blocking solution. After three washing steps appropriate Alexa conjugated secondary antibodies were subsequently incubated for 2 h at RT and cultures were washed, counterstained with DAPI and mounted in Fluoromount reagent.

### 2.10 Immunohistochemistry

8‐month‐old heterozygous APP/PS1‐21 and wild‐type male mice were killed by decapitation after deep isoflurane gas anesthesia. Their brain was rapidly dissected, fresh frozen, and serially cut at 10 μm thick using a cryostat prior to being processed for immunostaining. Briefly, coronal brain sections were fixed in 4% PFA for 10 min, washed twice in PBS and incubated for 1 h in a non‐permeabilizing buffer in PBS with 1% BSA. Subsequently, sections were incubated with the biotinylated BP at 50 nM in PBS‐BSA for 2 h at RT. Next, antibodies directed against biotin and Aβ_17‐24_ were applied overnight at 4°C prior to a 2 h incubation step with fluorescently conjugated secondary antibodies. After staining, sections were counterstained with DAPI prior to mounting in Fluoromount reagent.

### 2.11 Confocal acquisition and analysis

Confocal images of immunostainings were obtained using a confocal Zeiss LSM 800 or 880 AiryScan microscope equipped with two diode lasers (405, 561 nm), a laser gas argon (488 nm) and a laser gas He/Ne (633 nm), with a 63× objective and an additional zoom factor (3×). For image acquisition, identical parameters were used between genotypes or cell culture conditions by an investigator blinded to treatments. 3D confocal z‐stack images were acquired and for hippocampal cell images, deconvolved using Huygens software.

Subsequent analyses of hippocampal synapse staining were performed by a blinded investigator with Icy software. To assess biotinylated Aβ expression at full synapses, regions of interest (ROIs) used to identify synapses were defined as the colocalization area between PSD95 and Bassoon positive clusters on the dendrite of hippocampal neurons. Biotinylated Aβ clusters were quantified in each ROI in collapsed Z‐stacks. A published detailed version of the protocol is available following this link: https://icy.bioimageanalysis.org/protocol/amyloid‐clusters‐colocalisation‐on‐synapses/.

### 2.12 Transmission electron microscopy

Prior to imaging, 10 μL of preparation of Aβ fibrils (30 μM) aggregated for 24 h at 37°C in the presence or absence of equimolar concentrations of CP or BP were deposited onto Formvar‐coated mesh copper grids. After a 2 min incubation step at RT, grids were rinsed with sterile dH_2_O. Samples were subsequently stained using 2% uranyl acetate for 1 min in the dark to increase contrast and were allowed to air dry for 2 min. Finally, they were visualized with a Philips EM208 Transmission Electron Microscope operating at 120 kV, initially imaged at low magnification (20,000×), and thereafter at 120,000× using a Gatan Orius 600 digital camera.

### 2.13 Statistical analysis

Data were expressed as mean ± SEM in ThT, Western blotting, synaptic targeting experiments and electrophysiological recordings (Table S2). Normality and variance homoscedasticity were assessed using descriptive analysis and appropriate tests. Sample size (*n*) was determined based on previous studies from the literature and pilot experiments; n refers to the number of ELISA, ThT or Western Blotting experiments, to coverslips or culture wells for hippocampal neuron cultures, and to mice per condition or per genotype for electrophysiological experiments. For Aβ‐VEGF competitive ELISA experiments, IC_50_ was determined by fitting the data to a three‐parameter sigmoidal dose–response curve. For the analysis of Aβ‐BP interaction, *K*_D_ was determined by fitting the data to a two‐parameter saturation curve. For ELISA assays, Aβ + CP binding to VEGF or Aβ binding to CP were fitted using a linear model (R software). For quantitative assessment of Aβ aggregation, Aβ expression level and the percentage of synapses colocalized with Aβ, data were compared for statistical significance between groups using a Kruskal–Wallis and a Dunn's post hoc test with Holm correction (R software) (Table S3). For electrophysiological data, statistical analysis between treatments and/or genotypes were carried out using a Kruskal–Wallis followed by a Dunn's post hoc test to compare differences in fEPSP slopes (Table S3).

## 3 RESULTS

### 3.1 High‐affinity interaction of BP with Aβo

**FIGURE 1:**
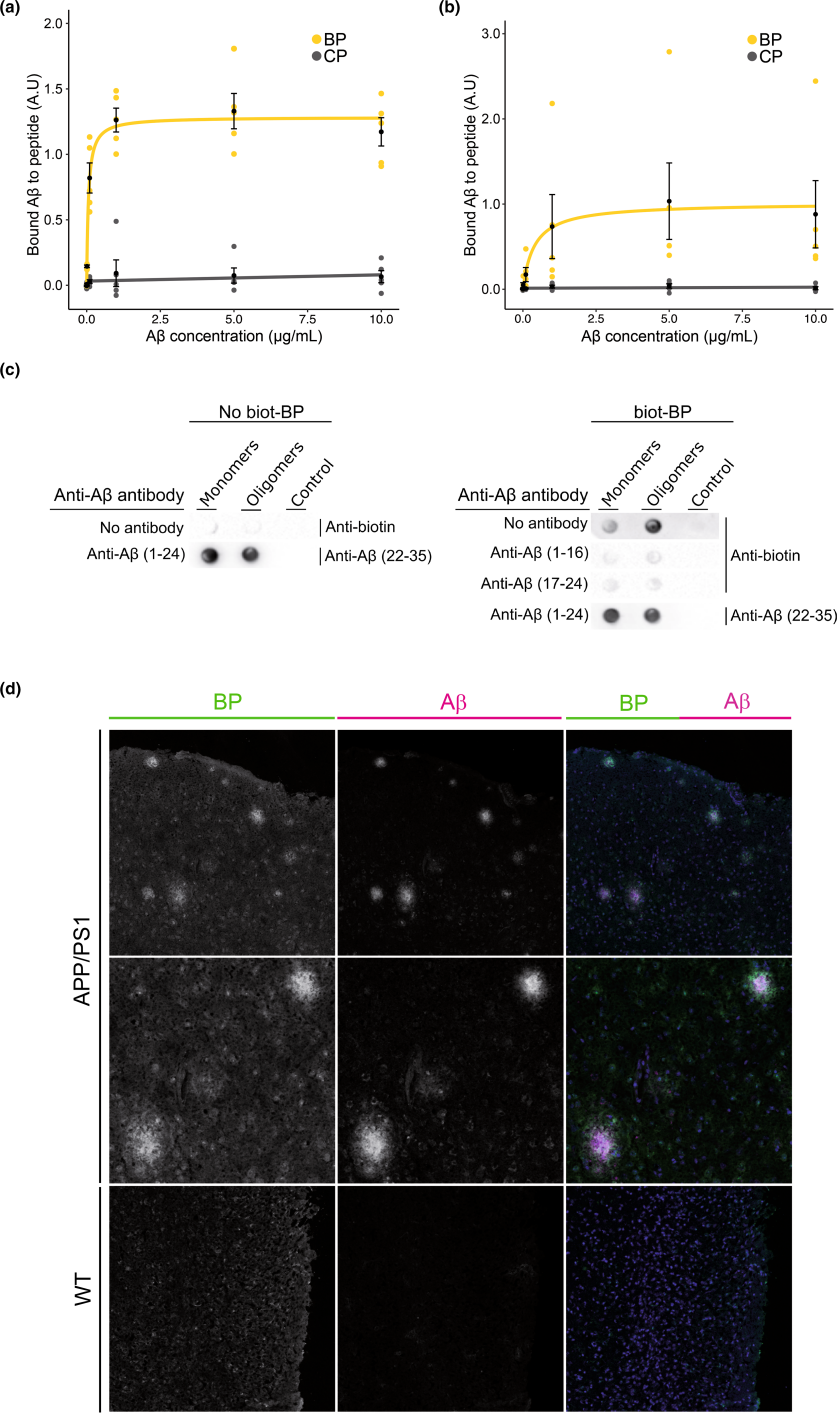
BP strongly binds to Aβo with high affinity. (a) Dose‐dependent binding of biotinylated Aβ_42_ oligomers to immobilized BP analyzed by ELISA, with a titration binding curve showing the strong binding ability of Aβo for BP. (b) ELISA assay illustrating the modest dose‐dependent binding of Aβ_40_ monomers to immobilized BP. Note that no binding of the CP is detected to either Aβo (a) or Aβm (b). *n* = 5–4 independent experiments, data represent mean ± SEM. (c) Epitope mapping of the interaction between Aβo and BP. On the left panel, Aβm and Aβo were immobilized on a nitrocellulose membrane and probed with the anti‐biotin antibody to control for non‐specific binding. The anti‐Aβ (22–35) specifically recognizes Aβm and Aβo after a preincubation step with anti‐Aβ (1–16) and (17–24) antibodies. On the right panel, immobilized Aβm and Aβo were then incubated for 2 h with the biotinylated BP (biot‐BP), and bound biot‐BP was recognized by the anti‐biotin antibody (top row). Note that the biot‐BP preferentially binds Aβo. The BP binding epitope on Aβo was identified by signal suppression in the presence of antibodies targeting Aβ (1–16) or Aβ (17–24) incubated prior to biot‐BP (middle rows). On the bottom row, co‐incubation with anti‐Aβ (1–16) and (17–24) was used to target Aβ (1–24) region prior to biot‐BP incubation. Anti‐Aβ (22–35) immunoblotting revealed that BP does not bind the Aβ (22–35) region. (d) Binding of biot‐BP to Aβ plaques and peri‐plaque area. Immunostainings of 8‐month‐old APP/PS1 and WT brain sections showing bound biot‐BP (left, green on the merge) in relation to Aβ plaques identified with anti‐Aβ immunostaining (middle, magenta on the merge). Note in the merge that biot‐BP staining is found in the plaque core and in the peri‐plaque surrounding the core (green halo). Scale bars, (d), 100 μm (top and low rows), 50 μm (middle row).

Taking advantage of the characterized interaction between VEGF and Aβo (Martin et al., 2021), we designed a new peptide mimicking part of the VEGF protein sequence, named the blocking peptide (BP), to specifically target Aβo. The BP is a 15‐amino acid synthetic peptide corresponding to the first heparin‐binding domain of the VEGF_206_ isoform. As a control peptide (CP), we produced a peptide encompassing the Flag sequence, which is not expected to bind Aβ. To determine the ability of BP or CP to bind Aβ and characterize their binding affinity, we performed an ELISA assay using increasing concentration of biotinylated Aβ_42_ oligomers (Aβo) or biotinylated Aβ_40_ monomers (Aβm) added to wells precoated with BP or CP. Preparations of Aβo enriched in low molecular species (LMW Aβo) (Martin et al., 2021) or Aβm were used at concentrations ranging from 0 to 10 μg/mL on immobilized CP or BP. Aβo (Figure 1a) or Aβm (Figure 1b) failed to bind to the CP even at the highest concentration, whereas Aβo exhibited a strong binding to BP, reaching a plateau at 1 μg/mL (Figure 1a). A saturation fitting curve equation and a Scatchard analysis were carried out to determine the Aβo binding affinity for BP, which was in the low nanomolar range (*K*_D_ = 12 nM). In contrast, the binding ability of the Aβm for BP was much weaker, reaching a plateau at 5 μg/mL (Figure 1b), with a low affinity and a *K*_D_ value of 19 μM. Thus, oligomeric species rich in LMW Aβo bind BP with high affinity. To characterize the BP binding sites on Aβ, we then performed an epitope mapping experiment using the biochemical dot blot approach. Aβm and LMW Aβo aggregated for 24 h at 4°C were immobilized on nitrocellulose membranes and probed with specific antibodies directed against the N‐terminal and central regions of Aβ. We reasoned that if an antibody interferes with the binding of biotinylated‐BP on Aβo, this would indicate that it recognizes the same epitope as the BP, or a very near neighbor epitope. Notably, the two antibodies targeting the N‐terminal (residues 1–16) and central hydrophobic core of Aβ (residues 17–24) abrogated the BP‐Aβo interaction (Figure 1c, Figure S1). In contrast, BP does not bind the aggregation‐prone region of Aβ (residues 22–35) (Figure 1c). Altogether, these findings revealed that the 1–24 epitope is the most critical region of Aβ for this interaction.

Having established that BP binds LMW Aβo with high affinity at specific epitopes, we investigated whether it can associate in situ with Aβ. Therefore, we incubated brain sections from the APP/PS1 mouse model of AD with biotinylated BP and identified bound‐BP and amyloid plaques using anti‐biotin and anti‐Aβ immunostainings, respectively. BP allowed the staining of amyloid plaques and in particular of their periphery at low concentration (Figure 1d), indicating its association with Aβ oligomers reported to be present as an halo surrounding the Aβ plaque core (Koffie et al., 2009).

### 3.2 BP disrupts Aβ aggregation and inhibits fibril formation

**FIGURE 2:**
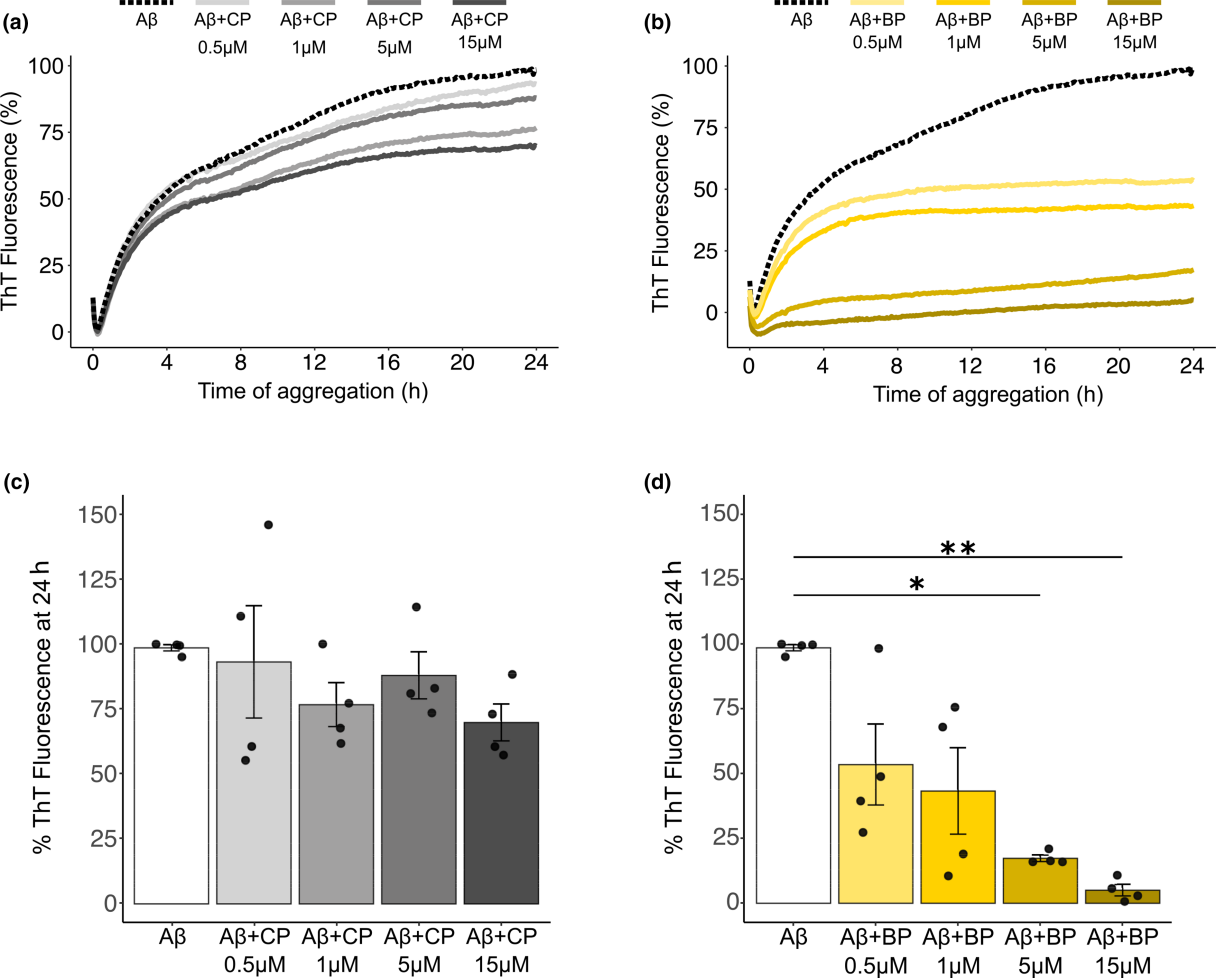
BP prevents Aβ fibrillation in a dose‐dependent manner. (a, b) Aggregation kinetics analyzed by ThT fluorescence using 15 μM of Aβm with increasing concentration of the CP (a) or BP (b). Data represented mean values of ThT fluorescence expressed as the percentage of maximum ThT fluorescence, *n* = 4 independent experiments. (c, d) Quantitative analysis of ThT fluorescence at 24 h, with the CP showing no significant effect on Aβ aggregation (c), in contrast to the BP efficiently preventing Aβ fibrillation in a dose‐dependent manner (d). Kruskal–Wallis, *p* = 0.0038, followed by Dunn post hoc test, *n* = 4 independent experiments. *p < 0.05; **p < 0.01.

One of the key pathogenic mechanisms in AD is the toxicity resulting from Aβ ability to self‐aggregate in species rich in β‐sheet‐based structure (Iadanza et al., 2018). Various aggregated assemblies are generated from Aβm during this aggregation process, from soluble Aβo in the early stages to insoluble fibrils (Stine et al., 2003). To determine whether BP could interfere with the Aβ aggregation process and Aβ fibrillation kinetics, we used a Thioflavin T assay (ThT). Thioflavin T is well known to give a strong fluorescent signal proportional to the amount of β‐sheets, when bound to amyloids (Xue et al., 2017). As expected, incubation of Aβ monomers at 15 μM induced a strong increase in ThT fluorescence, reaching a plateau after 16 h of incubation. Increasing concentrations of CP had no impact on ThT fluorescence compared to Aβ alone (Figure 2a,c). In contrast, BP substantially decreased the ThT fluorescence at the aggregation plateau, in a concentration‐dependent manner (Figure 2b). Importantly, quantitative analyses performed at 24 h revealed that BP significantly decreases Aβ fibrillation by 82 ± 1.24% at 5 μM, with a maximum effect obtained using a BP concentration equimolar to that of Aβ (95 ± 2.2%, *n* = 4, Figure 2d). Thus, BP successfully disrupted Aβ aggregation and inhibited fibrillation.

**FIGURE 3:**
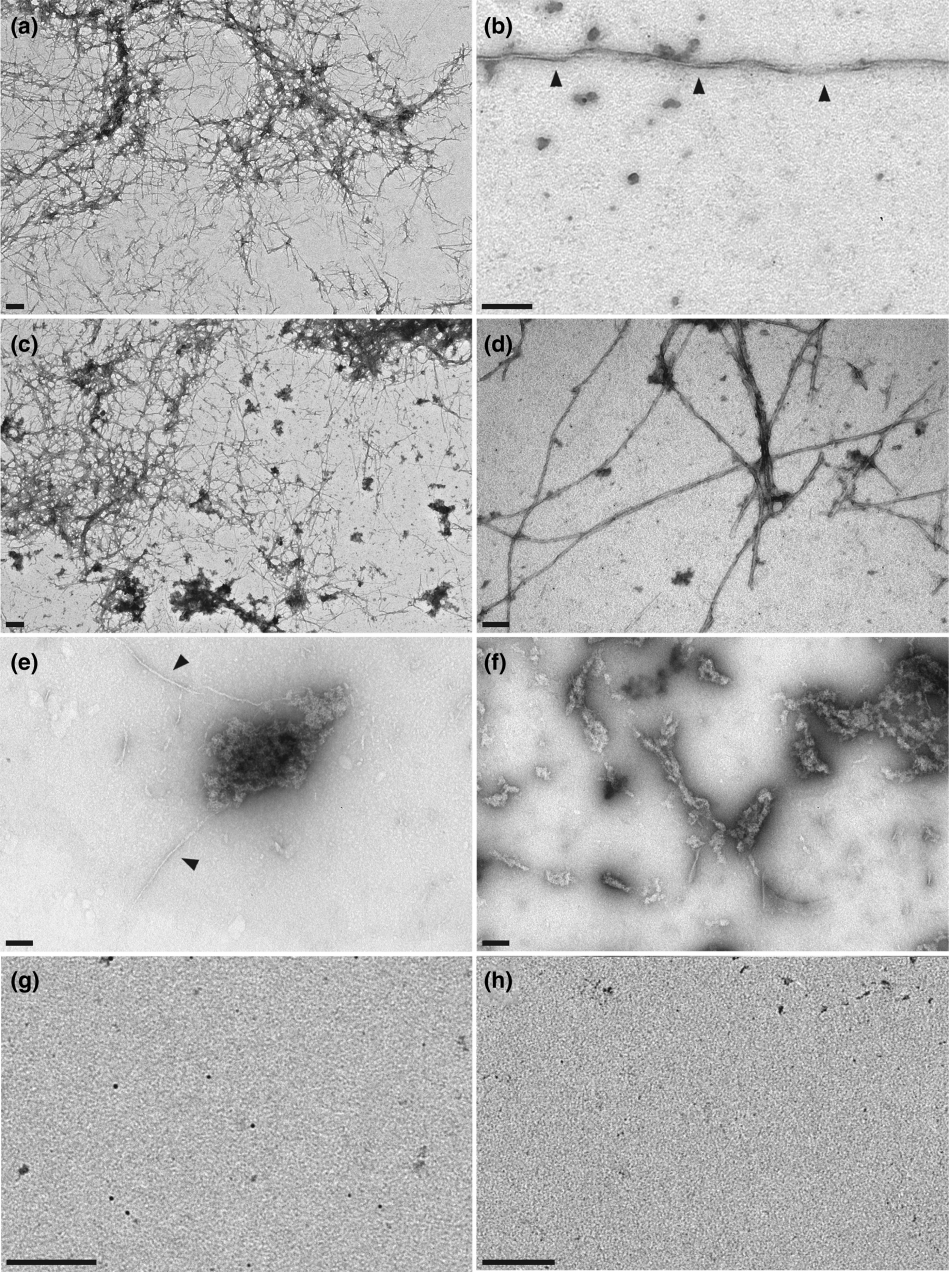
BP inhibits Aβ fibril formation and promotes amorphous aggregates. (a, b) TEM photomicrographs illustrating Aβ fibrils formed after 24 h aggregation in the Aβ condition (30 μM), with fibrils displaying a characteristic twisted morphology (b, black arrowheads). (c, d) Similar fibrillary structures were obtained when Aβ aggregated with equimolar concentration of the CP. (e, f) However, Aβ aggregates formed in presence of BP were amorphous in shape, with infrequent fibrillary structures (e, black arrowhead). (g, h) The CP (g) or BP (h) alone do not show any detectable 3D structures by TEM. Scale bars, (a, c), 200 nm; (b, d–h) 100 nm. *n* = 4 independent experiments.

To confirm the BP inhibitory effect on Aβ fibrillation and characterize the size and structure of Aβ‐formed aggregates, we performed transmission electron microscopy (TEM) experiments. Aβ was aggregated for 24 h at 30 μM, alone or in presence of CP or BP at equimolar concentrations. As expected, when aggregated alone or with CP, Aβ exhibited typical fibrillary structures with a characteristic twisted morphology (Figure 3a–d). In contrast, in presence of BP Aβ aggregation led to the formation of large amorphous aggregates associated with infrequent fibril structures (Figure 3e,f). Importantly, such amorphous aggregates were never observed in the Aβ or Aβ + CP conditions. Control experiments validated that neither CP (Figure 3g) nor BP (Figure 3h) induced 3‐dimensional structures. Altogether, our findings indicate that BP promotes the formation of amorphous Aβ assemblies that are deviating from the aggregation/fibrillation pathway and considered as less toxic (Lieblein et al., 2020).

### 3.3 BP prevents Aβ HMW oligomers formation

**FIGURE 4:**
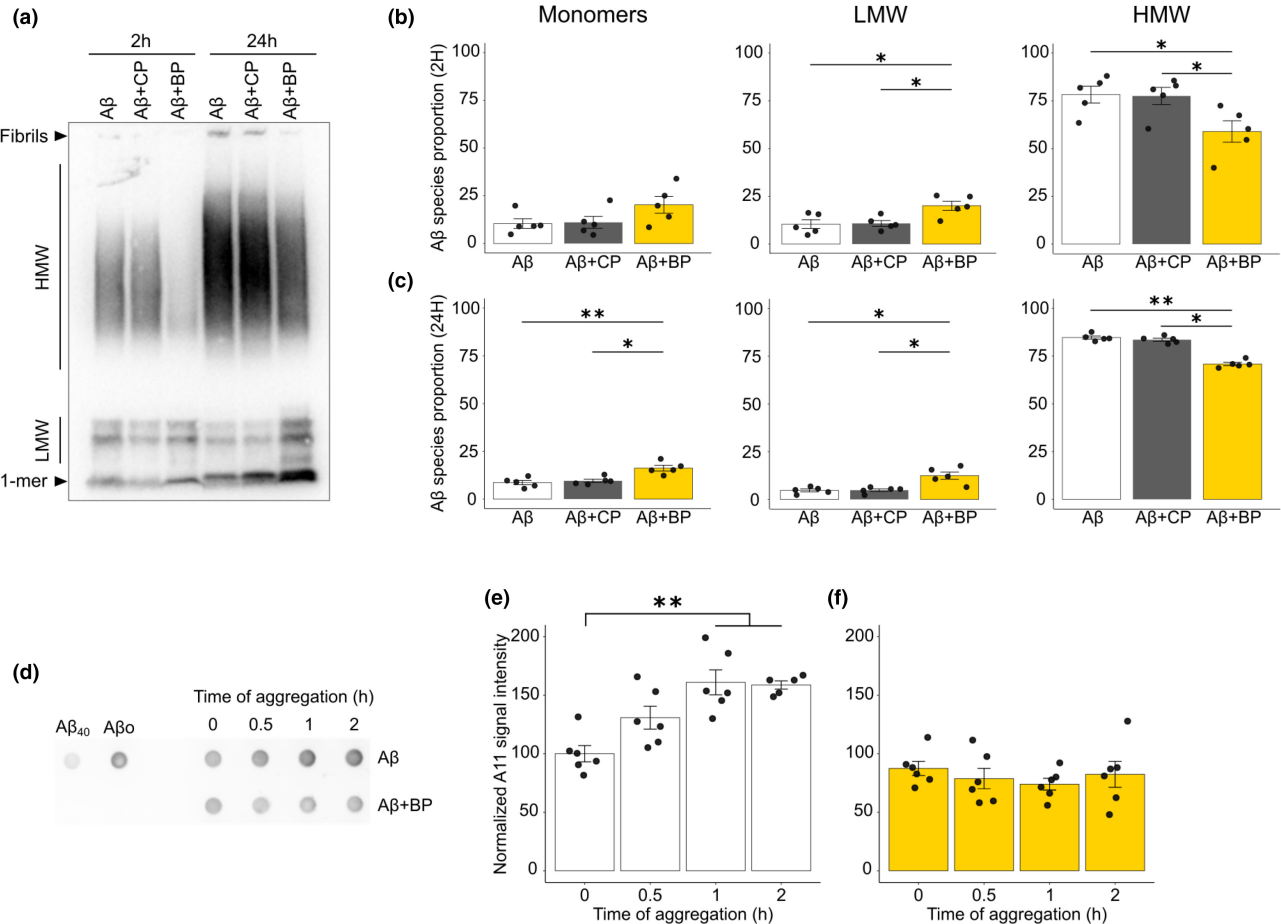
BP impedes the formation of HMW Aβo. (a) Representative Western Blotting experiment probed with the 6E10 anti‐Aβ antibody showing Aβ aggregated for 2 h or 24 h at 37°C, alone or in presence of equimolar concentration of CP or BP. Note the change in proportion of HMW, LMW Aβo and monomers (1‐mer) in the presence of the BP, but not with the CP. (b) Semi‐quantitative analyses of the resulting pool of soluble Aβ species at 2 and 24 h (c). The BP significantly decreases HMW Aβo fraction and increases that of LMW Aβo at 2 and 24 h, and Aβm at 24 h. Kruskal–Wallis followed by Dunn post hoc test, *n* = 5 independent experiments. (d) Representative dot blot probed with the conformational A11 antibody showing pre‐fibrillar Aβ aggregates when Aβ is incubated alone or in presence of the BP, from 0 to 2 h at 37°C. (e, f) Semi‐quantitative analysis of A11 signal intensity for Aβ alone (e) or Aβ + BP (f) condition, normalized to the signal obtained with Aβ alone at 0 h. Kruskal–Wallis followed by Dunn post hoc test, *n* = 5–6 independent experiments. *p < 0.05; **p < 0.01.

Unlike insoluble Aβ fibrils, smaller soluble aggregates, the Aβo, are now considered as the most bioactive and toxic Aβ species (Lue et al., 1999; McLean et al., 1999). Thus, it is critical to determine whether BP which prevents Aβ fibrillation could also affect the early stages of Aβ aggregation during which toxic prefibrillar Aβo are formed (Chiang et al., 2018). To characterize BP impact on the formation of soluble Aβ species, we incubated Aβm for 2 h at 37°C to enrich Aβ species in high molecular weight (HMW) Aβo, or for 24 h to favor larger Aβ aggregates. Aβm was incubated alone or in presence of equimolar concentrations of CP or BP, and the resulting pool of soluble Aβm, LMW and HMW Aβo was analyzed by Western Blot using the 6E10 anti‐Aβ antibody (Figure 4a). After 2 h incubation, BP significantly reduced the fraction of HMW Aβo from 78 ± 4.4% in the Aβo alone condition to 59 ± 5.6% in Aβo + BP condition (Figure 4b). This reduction was accompanied by an increase in LMW Aβo from 10.5 ± 2.3% to 20 ± 2.4% in the Aβo alone versus Aβo + BP condition (Figure 4b), whereas the CP did not change the proportion of Aβo species. A similar effect was obtained at 24 h, since BP also decreased the proportion of HMW Aβo compared to the Aβo condition (from 85 ± 0.8% to 71 ± 0.9%) with a combined increase in LMW Aβo (from 5 ± 0.8% to 12 ± 1.9%) and Aβm (from 8.6 ± 1.1% to 16 ± 1.5%) (Figure 4c). Notably, we also observed a decrease in the amount of fibrils at the top of the gel, in presence of BP, but did not take it into account in the quantitative analysis because fibrils cannot enter the gel. Taken together, these results demonstrate that BP selectively prevents the formation of HMW Aβo, but not that of smaller species. This selective inhibition of BP could result from its ability to bind small Aβo, which might prevent further assembly beyond LMW Aβo.

Since it has been suggested that LMW Aβo and Aβm identified by western blotting may result from the dissolution of larger aggregates (Bitan et al., 2003), we next used a native dot blot approach probed with a conformational antibody that specifically recognize soluble Aβo (Kayed et al., 2003). Notably, the A11 antibody has been shown to target a conformational structure common to toxic oligomeric proteins and block their toxicity in human neuroblastoma cells (Kayed et al., 2003). Thus, Aβm alone or in presence of BP was incubated from 0 to 2 h at 37°C, and pre‐fibrillar Aβ aggregates were analyzed using the A11 antibody. Dot blot assay demonstrated that Aβ oligomerization process produces an increasing amount of A11 immunoreactive Aβo species over time with a weak reactivity against Aβm (Figure 4d,e). In contrast, Aβ oligomerization performed in presence of BP does not form additional A11 binding species to those detected at the start of the incubation, even after 2 h of oligomerization (Figure 4d,f). These findings suggest that Aβ species produced with the BP originate from an alternative pathway giving rise to species with different conformational structures. This alternative pathway probably involves Aβm aggregation into unstructured or amorphous aggregates such as those identified by TEM.

### 3.4 BP interferes with Aβo synaptic targeting

**FIGURE 5:**
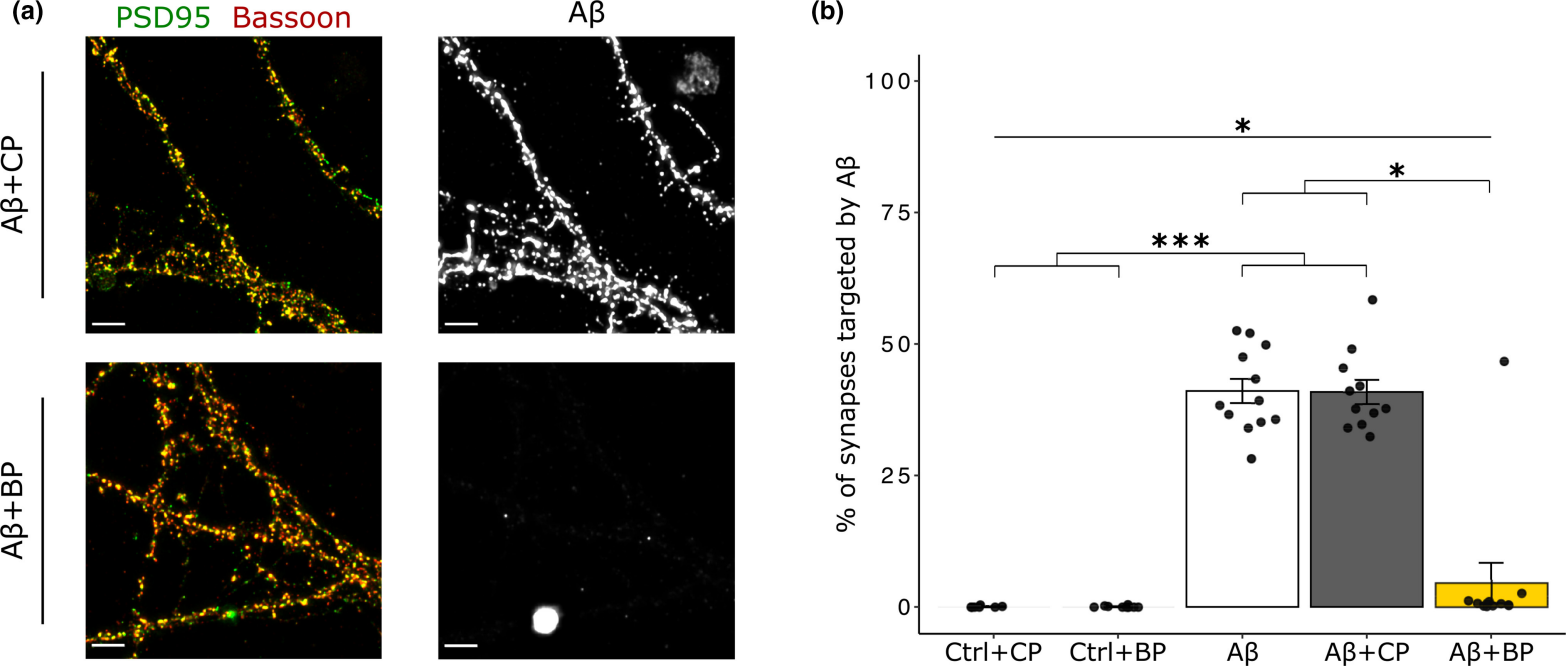
BP inhibits the formation of Aβo able to target synapses. (a) Confocal images of DIV21 hippocampal neuron cultures treated with biotinylated Aβ preincubated for 2 h at 37°C with CP or BP at equimolar concentrations. Full synapses (yellow clusters) were characterized by PSD95 (green) and Bassoon (red) marker colocalization, with neuronal cell surface Aβ binding shown in grey. Scale bar = 5 μm. (b) Quantitative analysis showing the percentage of Aβ‐bound synapses with a dramatic decrease in Aβo synaptic targeting in the Aβ + BP condition. Kruskal–Wallis (*p* = 1.9e−08) followed by Dunn post hoc test, *n* = 8–12 from 3 independent experiments, *p < 0.05; ***p < 0.001.

Extensive studies now indicate that Aβo induce a synaptic failure at early stages of AD by exerting their toxic effect as specific ligands for synapses (Colom‐Cadena et al., 2020; Esparza et al., 2013; Koffie et al., 2009). In hippocampal neurons, excitatory synapses have been shown to be prominently targeted by HMW Aβo over LMW Aβo (Lacor et al., 2004). Thus, to investigate the ability of HMW Aβo formed with or without BP to target synapses, we treated mature hippocampal neuron cultures at DIV21 for 30 min with 0.5 μM of biotinylated Aβo aggregated for 2 h in presence of CP or BP. Synapses were identified by the colocalization of Bassoon and PSD95, markers of pre‐ and post‐synaptic zones respectively, and Aβ immunoreactive clusters were detected with fluorescently labeled streptavidin. Aβo alone or in the presence of CP showed a strong binding to hippocampal neuron surface, which specifically concentrated at Bassoon and PSD95 positive synaptic sites. On the contrary, Aβo binding to neuron cell surface was strongly prevented when Aβ oligomerized in presence of BP, with little colocalization with synaptic clusters (Figure 5a). Quantitative analyses of Aβ‐bound synaptic sites indicated that around 40% of synapses were targeted by Aβ alone or with the CP (Figure 5b). Importantly, the percentage of synapses targeted by Aβo strongly and significantly decreased in the Aβ + BP condition to 4.6 ± 3.8%, showing a 89% reduction in Aβ binding to synapses that matched the values obtained in control conditions, without Aβ treatment (Figure 5b). Overall, these findings demonstrated that BP prevented the specific binding of Aβo to synapses, supporting the view that Aβo formed with BP are less bioactive, with a compromised toxicity.

However, the possibility that BP could act by competing for Aβ binding sites on synapses cannot be ruled out. Thus, to investigate whether BP may impede Aβo binding to synaptic partners, we treated hippocampal neuron cultures with pre‐formed Aβo and BP for 30 min. Confocal imaging revealed a strong Aβ binding to synapses in all Aβo treated conditions with no effect of BP (Figure S2A), which was confirmed by the quantitative analysis (Figure S2B). Collectively, our results emphasize that BP impedes the formation of bioactive Aβo that are no longer able to bind synapses, and further suggest that the underlying mechanism does not involve BP‐mediated blockade of putative Aβ receptors.

### 3.5 BP rescues LTP alterations in APP/PS1 mice

Compelling evidence demonstrated that soluble Aβo from various sources cause a robust inhibition of the Long‐Term Potentiation (LTP), a cellular correlate for learning and memory (Mucke & Selkoe, 2012). Given that BP inhibited the formation of Aβ oligomers that target synapses, we wondered whether it could have an impact on LTP.

Prior to LTP experiments, we explored whether BP exerts an effect on synaptic function in hippocampal neurons using electrophysiological recordings in acute hippocampal slices from WT mice, and characterized potential changes in basal synaptic transmission at various BP concentrations. BP was administrated to slices over a range from 0.3 to 2 μM for 30 min and changes in basal synaptic transmission were reflected as changes in EPSP slope. A significant reduction in synaptic transmission was observed at 2 μM but did not occur at lower concentrations, such as at 0.5 μM (Figure S3A,B). Thus, we selected the 0.5 μM concentration to explore the acute effect of BP on LTP inhibition in the APP/PS1 mouse model of AD.

**FIGURE 6:**
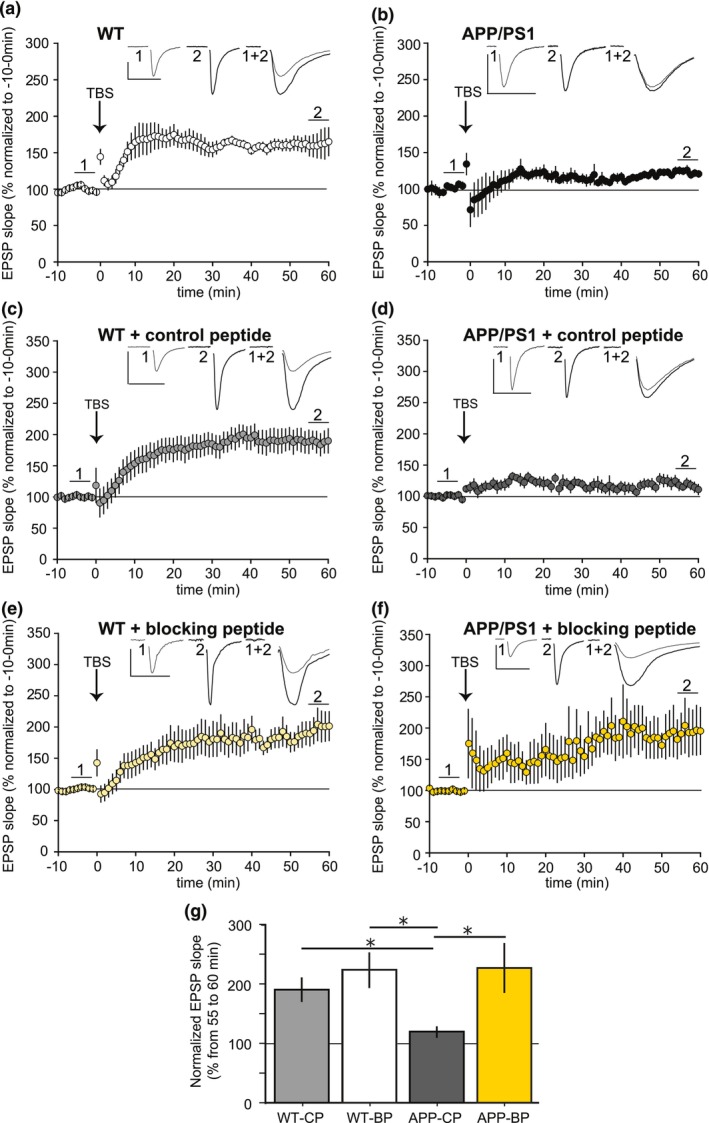
Restoration of LTP in aged APP/PS1 mice slices after BP treatment. (a–f) Time course of synaptic responses at the Schaffer collateral to CA1 synapses after inducing LTP with a Theta Burst Stimulation protocol (TBS) in slices from 8‐month‐old WT (a, c, e) and APP/PS1 mice (b, d, f). Representative fEPSP traces (average of 5 min recording immediately before TBS (1, thin line) and 55 min after TBS (2, thick line). Traces 1 + 2 represent superimposed traces 1 and 2 with a four‐fold stretch of their time axis to better illustrate the fEPSP slopes. A robust LTP was induced in WT slices (a, *n* = 6 mice) whereas an impaired LTP was recorded in APP/PS1 slices after TBS (b, *n* = 7 mice). The incubation of CP (c, *n* = 6 mice) or BP (e, *n* = 6 mice) at 0.5 μM at least 40 min before TBS did not modulate LTP in WT slices. In APP/PS1 slices, although CP failed to induce an LTP after TBS induction ( d, *n* = 7 mice), BP treatment restored a robust LTP response 60 min after TBS induction (f, *n* = 7 mice). (g) Summary bar graph showing that LTP is significantly decreased in APP/PS1 slices incubated with the CP compared to CP‐treated WT slices (LTP amplitude computed as fEPSP slope 55–60 min after TBS is decreased from 191.4 ± 18.3% in WT slices + CP to 119 ± 9.1% in APP/PS1 slices + CP, *n* = 7–6 mice, Kruskal–Wallis test *p* = 0.01, *χ* ^2^ = 10.73, df = 3, Dunn post hoc test *p* < 0.05). In contrast, BP treatment rescued LTP in APP/PS1 slices compared to the CP treatment (LTP is increased from 119.8 ± 9.1% in APP/PS1 slices + CP to 226.7 ± 40.5% in APP/PS1 slices + BP, *n* = 7–7, *p < 0.05).

We investigated the effect of acute BP treatment in hippocampal slices derived from 8‐month‐old APP/PS1 mice, which have been shown to display strong LTP alterations (Gengler et al., 2010). CA1 field Excitatory Post Synaptic Potentials (fEPSP) were recorded in acute slices from WT and APP/PS1 mice before and after Theta Burst Stimulations (TBS) of Schaffer Collaterals (SC). As expected, TBS elicited a robust LTP at SC‐CA1 synapses in WT slices, but failed to induce an LTP in APP/PS1 slices even 60 min after TBS (Figure 6a,b), in agreement with a previous study (Gengler et al., 2010). We then incubated WT or APP/PS1 slices with CP or BP at 0.5 μM, at least 40 min before TBS induction. Importantly, CP treatment failed to restore LTP after TBS in APP/PS1 slices (Figure 6d), whereas BP successfully rescued LTP, which persisted for at least 60 min after TBS (Figure 6f). Notably, neither CP nor the BP impacted LTP responses in WT slices (Figure 6c,e), indicating that BP has no effect on LTP in the absence of Aβ aggregates. Quantitative analyses of fEPSP slopes demonstrated that LTP was strongly and significantly impaired in APP/PS1 slices treated with the CP compared to CP‐treated WT slices (119 ± 9.1% in CP‐treated APP/PS1 slices versus 191.4 ± 18.3% in CP‐treated WT slices, post hoc test, *p* < 0.05; *n* = 7–6, Figure 6g). In contrast, BP treatment substantially alleviated LTP impairment in APP/PS1 slices to reach LTP amplitude obtained in WT slices (226.7 ± 40.5% in BP‐treated APP/PS1 slices versus 223.8 ± 29.3% in BP‐treated WT slices, post hoc test, ns; *n* = 7–6, Figure 6g).

To better understand the underlying mechanism involved in LTP rescue triggered by BP in APP/PS1 slices, we examined the paired‐pulse facilitation induced by two electrical stimuli close in time (50 ms), a phenomenon known to be of presynaptic origin. The paired‐pulse ratio (PPR, see Section 2.5) was assessed before and after TBS in slices from the different experimental groups. A slight decrease in PPR was observed in both WT and APP/PS1 slices instantly after TBS, but no differences were detected between groups after CP or BP application (Figure S4). Thus, these findings indicate that the restoration of LTP in BP‐treated APP/PS1 group was not due to an increased probability of glutamate release at SC‐CA1 synapses, involving a presynaptic mechanism.

Furthermore, we explored whether BP effect on LTP was long lasting by stimulating two independent synaptic inputs onto the same CA1 neuronal population in WT and APP/PS1 hippocampal slices. TBS was applied to the S1 pathway (Stim 1) to elicit late‐LTP, whereas control synaptic responses were recorded from the independent S2 pathway (Stim 2) (Figure S5A), as previously reported (Navakkode et al., 2021). Importantly, BP treatment restored the late phase of LTP in APP/PS1 slices up to 180 min, without affecting the control pathway (Figure S5C, 261.8 ± 45.2% for the S1 pathway versus 98.6 ± 5.2% for the control pathway, *p* = 0.015, *n* = 5 mice).

Taken together, the present findings demonstrated that BP has the ability to rescue early and late LTP deficits in the APP/PS1 mouse model of AD, at an age when functional and structural synaptic alterations have already largely occurred.

### 3.6 BP effect on VEGF/VEGFR2 pathway and its alteration due to Aβo

Our previous report has shown that VEGF has the ability to preserve synapses from dysfunction and LTP deficits caused by Aβo (Martin et al., 2021). Consistent with this VEGF effect on LTP restoration, neuronal expression of VEGFR2 was shown to be mandatory in the hippocampus for allowing normal LTP expression. Thus, because BP corresponds to a part of the first Heparin Binding Domain (HBD) of the VEGF linear sequence (Martin et al., 2021), we investigated whether it could have an impact on VEGFR2 activation induced by VEGF. HUVEC cells naturally expressing VEGFR2 were treated with VEGF at 5 ng/mL with or without 0.5 μM of CP or BP for 5 min. Western Blotting analyses showed that acute application of BP or CP alone did not affect basal VEGFR2 phosphorylation level. Furthermore, they indicated that VEGF application induced a significant increase in VEGFR2 phosphorylation, which was not affected by BP or CP treatment (Figure S6A,B). Thus these data revealed that BP does not inhibit VEGFR2 signaling. We then investigated by ELISA assay whether BP could prevent the interaction between Aβo and VEGF that we characterized in our previous report (Martin et al., 2021). Increasing concentrations of CP preincubated with Aβo at 2.1 μM did not affect Aβo binding to immobilized VEGF, even at the highest concentration. In contrast, BP efficiently prevented Aβo binding to VEGF in a concentration‐dependent manner (Figure S6C). A sigmoidal dose–response curve fitting was used for data analyses which determined an EC_50_ of 350 nM in the BP condition. These results showed that BP can efficiently prevent Aβo‐VEGF interaction and potentially release VEGF.

## 4 DISCUSSION

In the present study, we demonstrated that the blocking peptide (BP) mimicking part of the heparin binding domain of the VEGF protein sequence inhibits the process of Aβ self‐aggregation, leading to the blockade of synaptic targeting by soluble Aβ‐derived oligomers (Aβo). Using a combination of biochemical, cell imaging, and electrophysiological approaches, we deciphered the mode of action of BP on molecular and cellular mechanisms underlying AD pathogenesis. First, we revealed that BP directly interacts with Aβo enriched in LMW species with a high affinity in the nanomolar range. Second, we further revealed that this direct interaction impedes Aβ aggregation process, with an impairment in the formation of HMW Aβo at early stages, which results ultimately in fibril formation blockade. Consequently, Aβo formed in the presence of the BP failed to display reactivity to the A11 conformational antibody, which has been reported to recognize highly toxic Aβo. Furthermore, Aβo assemblies formed with the BP lose their ability to bind to synapses, consistent with the protecting role of the BP against the disruption of synaptic plasticity observed in hippocampal slices from the APP/PS1 mouse model of AD. Together these data provided the first evidence of a key role for a VEGF‐derived peptide in blocking the toxic Aβ aggregation process and maintaining synaptic function in AD pathology.

### 4.1 Impact of the BP on Aβ aggregation

In AD, a key pathological process is the self‐aggregation of Aβm into β‐sheet‐rich Aβo species (Lee et al., 2017). Initially, Aβo are derived from a pool of unstructured Aβm and proceed into larger β‐sheet‐rich soluble aggregates though the primary nucleation process, that ultimately leads to mature amyloid fibrils (Knowles et al., 2014; Lee et al., 2017). However, it is the fibril‐catalyzed secondary nucleation process that speeds up Aβ oligomerization and substantially increases the amount of Aβo (Cohen et al., 2013). Our kinetics study of Aβ aggregation using the ThT assay monitored the amount of β‐sheet amyloid structures over time and demonstrated a dose‐dependent inhibition of Aβ fibril formation in the presence of the BP. These findings imply that the inhibition in fibril formation induced by BP results from molecular interactions between BP and Aβ assemblies early in the aggregation process. However, we detected using an ELISA assay a low binding affinity of the BP to Aβm, in sharp contrast to its high affinity for LMW Aβo species, formed at 4°C for 24 h, which is in the nanomolar range. Thus, we speculated that BP preferentially binds to small LMW Aβo, preventing them to further associate in β‐sheet‐rich aggregates and fibrils. Determination of Aβo structure is challenging due to their transient nature and their high self‐aggregation rate, but transmission electron microscopy remains a benchmark technique to characterize the morphology of insoluble aggregates (Gremer et al., 2017). Our data revealed that BP promotes the formation of large amorphous Aβ aggregates after 24 h at 37°C, suggesting a diversion of soluble Aβo assemblies from the fibril forming pathway.

What are the implications of amorphous aggregates in AD pathophysiological events? Diffuse plaques with amorphous Aβ deposits and little or no fibrillar Aβ have not been associated with synapse loss or glial responses in AD brains, in contrast to dense‐core plaques rich in β‐sheet structures that are surrounded by dystrophic neurites and reactive glial cells (Serrano‐Pozo et al., 2011). Indeed, diffuse plaques are typically observed in the brain of non‐demented elderly people with cognitively intact abilities (Malek‐Ahmadi et al., 2016).

### 4.2 BP prevents conformational changes of Aβ oligomers linked to their toxicity

Convincing evidence now indicate that soluble Aβo rather than insoluble fibrils are the main culprit for triggering the first toxic effects on synapses in AD (Ashe, 2020; Sengupta et al., 2016). Thus, it is critical to understand how oligomers can induce toxicity and whether there is a structure‐toxicity relationship. Aβo species have been shown to display an increase in β‐sheet content as oligomerization proceeds with the formation of dimers, trimers, tetramers and so on, in contrast to Aβm that are largely unstructured (Ono et al., 2009). This increase in β‐sheet content is reflected at the ultrastructural level by a transition from thread‐like shaped monomers to spherical Aβo (Ono et al., 2009). Aβo have a profound impact on lipid bilayers by triggering lipid dispersion and extraction, whereas Aβm do not alter lipid membrane integrity (Bode et al., 2019). Similarly, cell membrane disruption has been reported for synuclein oligomers that are rich in β‐sheet structures which create a rigid core penetrating the membrane and impeding its integrity (Fusco et al., 2017). Thus, β‐sheet structures appear to be a key molecular basis of the oligomer‐mediated cell toxicity. As we demonstrated using TEM that BP promotes Aβ aggregation in amorphous assemblies devoid from β‐sheet structures, BP could stimulate an alternative pathway stabilizing unstructured Aβo. Interestingly, amorphous Aβo have been shown to be non‐toxic due to the loss of well‐defined β‐sheet structure (Ehrnhoefer et al., 2008) that provide Aβo the ability to bind to cell membranes and/or create calcium ion‐permeable channels or pore structures (Lee et al., 2017), as those formed with annular Aβ aggregates (Lashuel et al., 2002). To better characterize the impact of BP on toxic Aβo formation, we took advantages of reactivity of the A11 conformational antibody that recognizes an anti‐parallel β‐sheet structure rather than sequence‐based epitope which are common to toxic oligomeric proteins (Kayed et al., 2003; Laganowsky et al., 2012). As A11 antibody is known to target prefibrillar Aβo that are formed by primary nucleation before fibril formation (Ashe, 2020; Kayed et al., 2010), we investigated Aβ oligomerization kinetics with A11 reactivity as a readout of Aβo potential toxicity. Using non‐denaturing dot blot assay, we showed that there is a progressive and significant increase in A11‐immunoreactive toxic Aβo during the oligomerization process, as reported previously (Henning‐Knechtel et al., 2020), whereas BP blocks this time‐dependent increase in A11 binding to Aβo. Thus, BP may efficiently block the formation of toxic β‐sheet structured Aβo in favor of unstructured Aβo, not recognized by the A11 conformational antibody, emphasizing the potential protective role of the BP on Aβo toxicity.

### 4.3 Consequences of BP treatment on synaptic targeting by Aβ oligomers

Extensive research demonstrated that Aβo toxicity on synapses involves their selective binding to excitatory synapses (Mucke & Selkoe, 2012; Peng et al., 2022). High‐resolution imaging techniques and single particle tracking have revealed their unique ability to target synapses in living hippocampal neurons, whether they are derived from patient's brain with AD or from synthetic preparations (Lacor et al., 2004, 2007; Renner et al., 2010). Characterizing Aβo species that specifically bind synapses is challenging because time, ionic strength, temperature, concentration, Aβ isoform and pH influence Aβ association kinetics (Stine et al., 2003). In addition, time, concentration and Aβo nature have been shown to critically impact their synaptic targeting ability because synthetic HMW Aβo with a MW > 50 kDa predominantly bind synapses in a short time sequence of 30 min, over LMW Aβo (Lacor et al., 2004). Thus, we first investigated whether BP can affect the size of Aβo species during the oligomerization process and revealed that it substantially reduces the pool of HMW Aβo > 50 kDa over the LMW Aβo. The significance of HMW Aβo is particularly relevant since they have been reported to be the most abundant in the brain of AD patients (Hong et al., 2018; Yang et al., 2017) and could constitute reservoirs of toxic species (Yang et al., 2017). In the present study, biochemically characterized HMW Aβo have been shown to target more than 40% of excitatory hippocampal synapses identified by the co‐expression of the pre‐ and postsynaptic markers Bassoon and PSD95, in agreement with previous reports (Lacor et al., 2004). In contrast, only 5% of hippocampal synapses display Aβ binding, when Aβ is oligomerized in the presence of the BP. Thus, our findings demonstrated that BP which prevents the formation of HMW Aβo over LMW species, significantly impedes Aβo binding to synapses. Overall, they indicate that BP may have an important role in limiting the accumulation of Aβo at synaptic sites, raising the question of its impact on Aβo‐mediated synaptotoxicity.

### 4.4 Impact of BP on synaptic plasticity in the APP/PS1 mouse model of AD

At synapses, an increasing number of proteins have been identified as potential receptors for Aβo (Smith & Strittmatter, 2017), but the Aβo structure and size required to bind these receptors is still unknown. These specific and high affinity receptors are essential to the mechanism of Aβo toxicity since Aβo accumulation at both sides of the synapse causes major impairments in synapse function (Koffie et al., 2012; Pickett et al., 2016; Ting et al., 2007). Notably, Aβo isolated from soluble fractions of AD human brain extracts, from mouse or cellular models of the disease or derived from synthetic preparations, have been shown to be bioactive and to consistently inhibit long‐term potentiation (LTP), an electrophysiological correlate of learning and memory (Lambert et al., 1998; Shankar et al., 2008; Walsh et al., 2002). Here, we used LTP electrophysiological recordings as a readout for real time monitoring of Aβ‐induced alterations in synaptic function. We confirmed that theta burst stimulation (TBS) failed to induce LTP in the CA1 region in hippocampal slices derived from 8‐month‐old APP/PS1 mice, as previously reported (Gengler et al., 2010). These LTP alterations were detected long after the first structural changes of CA1 hippocampal synapses were reported in 3‐month‐old APP/PS1 mice (Paumier et al., 2022). Despite these early alterations, we demonstrated that acute BP treatments in 8‐month‐old APP/PS1 hippocampal slices result in the restoration of LTP responses within 1 hour after the TBS. The kinetics underlying the rapid rescue action of the BP suggest that BP could prevent the interaction between toxic Aβ species and synaptic receptors, and raises the question of whether presynaptic or postsynaptic mechanisms are involved. As we found no changes in the paired‐pulse ratios due to BP treatment that would likely indicate a presynaptic mechanism, we speculated that BP could modulate the activity of postsynaptic glutamate receptors in APP/PS1 hippocampal CA1 neurons. Interestingly, BP is a VEGF‐derived peptide and we previously reported that VEGF plays a key role in rescuing Aβo‐mediated impairments in hippocampal LTP, by maintaining the pool of postsynaptic AMPA receptors (Martin et al., 2021). In addition, we also highlighted the relevance of VEGFR2 receptor activity for adult hippocampal LTP (De Rossi et al., 2016) and revealed that VEGFR2 activation is blocked by Aβo and their interaction with VEGF (Martin et al., 2021). Therefore, we hypothesized that BP could limit VEGF trapping by interacting with Aβo assemblies in APP/PS1 hippocampal slices and allow VEGFR2 activation in CA1 neurons and LTP rescue. In support of this hypothesis, we further demonstrated that BP has the ability to prevent Aβo‐VEGF interaction.

In conclusion, our findings have major implications for pathogenic mechanisms in AD since the blocking peptide binds to specific populations of Aβ oligomers, interferes with their toxic aggregation process and further impedes the formation of Aβo that prominently target synapses. Furthermore, it specifically neutralizes Aβ‐mediated synaptotoxicity in a more complex AD model and rapidly normalizes synaptic plasticity processes that are a cellular correlate of learning and memory processes. Thus, we envision to use a chronic intranasal delivery system in vivo in APP/PS1 mice to overcome the Blood Brain Barrier and target brain Aβo with BP. Specific nanoformulations have already been shown to allow an efficient brain targeting with drug accumulation, due to their nanosize and chemical protective nanoenvironment (Agrawal et al., 2018). This would provide a strategy for assessing BP efficacy in preventing cognitive impairment and reducing Aβ load, and in limiting the development of neuritic plaques with related tau pathology in AD transgenic mice. Therefore, the present study opens the possibility of using a novel therapeutic option for reducing amyloid seeding and neutralizing highly toxic soluble Aβ oligomers in AD.

## AUTHOR CONTRIBUTIONS

PB and PdeG contributed equally to this work. PB and PdeG were involved in conception and study design, conducted experiments, analyzed and discussed data. PB was also involved in manuscript writing. MA performed experiments, analyzed and interpreted data. NC carried out culture experiments. JGD and JH discussed data and JH provided financial support. PAS contributed to study design, conducted electrophysiological experiments, analyzed and discussed data. CM conceptualized and supervised the study, wrote the manuscript and provided financial support. All authors commented on the manuscript.

## CONFLICT OF INTEREST STATEMENT

The authors declare no conflict of interest.

## Supporting information


Appendix S1

Appendix S2

## Data Availability

The data that support the findings of this study are available in the supplementary material of this article (Tables S2 and S3).
