# Supplementary material for: A novel peptide derived from vascular endothelial growth factor prevents amyloid beta aggregation and toxicity
Source: Aging Cell. 2023 Jul 6;22(9):e13907. doi: 10.1111/acel.13907 (PMC10497828; doi:10.1111/acel.13907)
Supplement: Supplementary file 1 — Appendix S1 [file ACEL-22-e13907-s001.pdf]

## Supporting Materials and methods

### Electrophysiological recordings

Field potential recordings were performed on adult wild-type mice to determine BP effect on basal synaptic transmission as a function of BP concentration at the Schaffer collaterals-CA1 synapses. Two-pathway stimulation experiments were performed for electrophysiological field potential recordings on hippocampal slices derived from 9-month-old wild type and transgenic heterozygous *APP/PS1-21* male mice. After baseline recordings of synaptic activity evoked at 0.2 Hz for at least 10 min, slices were treated with BP at 0.5 μM for 30 to 40 min before eliciting LTP with theta burst stimulation (TBS). TBS consisted of 10 trains separated by 30 s, each train composed of 10 bursts at 5 Hz and each burst providing 4 pulses at 100 Hz. Schematic representation of the experimental design is illustrated in Figure S5A: The recording electrode and the 2 stimulating electrodes were located in the stratum radiatum in CA1 to stimulate two independent pathways. TBS was applied to the stimulating electrode 1 (stim1) to evoke LTP in S1 pathway, whereas stimulating electrode 2 (stim2) elicited synaptic responses acting as a control pathway. Notably, a transient depression of fEPSPs was observed after TBS in the control pathway (S2).

### Cell cultures and treatments

*Hippocampal neurons*

Primary hippocampal cell cultures prepared from E17-18 C57Bl6/6JRj mice were used after 21 DIV for synaptic targeting experiments using LMW Aβo. Prior to treatments, LMW Aβo preparations were incubated at 5 μM for 1h at RT with or without CP or BP at equimolar concentrations. Cultures were then treated for 30 min with a final concentration of 500 nM biotinylated Aβ preparations enriched in LMW Aβo.

*HUVEC*

Human umbilical vein endothelial Cells (HUVEC) were obtained from Promocell and cultured in 6 cm Petri dishes pre-coated with collagen I at 5 μg/cm$^2$ in 20 mM glacial acetic acid. HUVEC were cultured at 7000 cells/cm$^2$ in endothelial cell growth medium MV2 heparin free (Promocell) supplemented with 1% P/S until reaching 80% confluency, and were used for up to 6 passages. The day before treatment, cell medium was replaced by a VEGF-deprived

medium. Cells were then treated for 5 min with VEGF (5 $ng.mL^{-1}$) with or without BP or CP at 0.5 µM. After treatments, HUVEC were washed and incubated in 600 mL of lysis buffer (Tris HCL 25 mM, EDTA 5 mM, DOC 0.5 mM, NP40 1%, SDS 0.1%, NaCl 150 mM), supplemented with cOmplete protease inhibitor cocktail 2%, Benzonase 0.02%, Orthovanadate 2%, and phosphatase inhibitors 2% for 10 min at 4°C. Cell lysates were then centrifuged at 1000 g for 10 min at 4°C to remove cellular debris, and stored at -20°C until use.

**Western blotting**

For immunoblotting of HUVEC cell lysates, 20 µg of proteins were separated on 3-8% gradient SDS-PAGE gels at 120V, with cathode and anode containing XT Trycine buffer. Gels were transferred on 0.2 µm nitrocellulose membranes at 120V for 2h in 5% methanol, Tris-Glycine buffer and immunoblotted. Anti-phospho-VEGFR2 (Tyr1175) and anti-VEGFR2 antibodies were used in combination with appropriate secondary antibodies (Table S1)

**Competitive ELISA assays**

Competitive ELISA experiments were performed to analyse the ability of the BP to impede biotinylated LMW Aβo binding to VEGF. For these assays, recombinant human $VEGF_{165}$ (500 $ng.mL^{-1}$) was immobilized in the 96-well microplate as the capture antigen and incubated overnight at 4°C. LMW Aβo were incubated at 10 $µg.mL^{-1}$ (2.1 µM) with various concentrations of CP or BP ranging from 10 nM to 100 µM for 1h at RT, and then added in triplicate on the plate after 3 washes with PBS and 0.05% Tween 20. The mix was incubated for 2h at RT, followed by a washing step, an incubation with the HRP-conjugated streptavidin for 20 min, additional rinses and finally the substrate reagent containing TMB and $H_2O_2$. After adding the stop solution, absorbance was measured using a TECAN microplate reader.

## SUPPORTING FIGURE LEGENDS

**Figure S1: BP prominently interacts with Aβo at a specific binding site.** Quantitative analysis showing a competitive interaction with 6E10 and 4G8 antibodies targeting the Aβ (1-16) and Aβ (17-24) epitopes respectively, that significantly blocks the binding of BP to Aβm (left panel) and Aβo (right panel). For Aβm, Kruskall-Wallis, p = 0,0086, n = 5 independent experiments; pairwise Wilcoxon rank sum post-hoc test *p<0,05; for Aβo, Kruskall-Wallis, p = 0,0081, n = 5 independent experiments; pairwise Wilcoxon rank sum post-hoc test *p<0,05). Data represents mean ± SEM.

**Figure S2: BP does not act by competing for Aβo binding sites on synapses. A)** Confocal images of DIV21 hippocampal neuron cultures treated for 30 min with biotinylated pre-formed Aβo pre-incubated at RT with CP or BP at equimolar concentrations. Full synapses (yellow clusters) were characterized by PSD95 (green) and Bassoon (red) marker colocalization, with neuronal cell surface Aβo binding shown in grey. Scale bar = 5 µm. **B)** Quantitative analysis illustrating the percentage of Aβ-bound synapses with no effect the BP compared to the other Aβ- treated conditions. Kruskal Wallis (p = 8.8e-06) followed by Dunn post-hoc test, ns = not significant, n = 6-11 from 2 independent experiments.

**Figure S3: BP impact on synaptic transmission. A)** Time course of BP effect on fEPSP at the Schaffer collateral to CA1 synapse in WT mice. Although the application for 30 min of 2 µM BP induces a significant decrease in the fEPSP slope (Wilcoxon, p<0.02, n = 6), a concentration of 0.5 µM has no effect (ns, n = 11). Right: the bar graph shows a significant difference in the synaptic slope measurement between the two BP concentrations (Mann-Whitney, p<0.02, n = 6-11 mice. **B)** Dose-response curve showing BP effects on basal synaptic transmission (normalized to baseline responses before BP application). A logistic curve was used to fit fEPSP slopes as a function of BP concentrations (adjust r2 = 0.79, p = 0.002).

**Figure S4: Similar paired-pulse facilitation level in BP and CP-treated APP/PS1 and WT slices.** Time course of paired-pulse ratio at the Schaffer collaterals to CA1 synapses after a Theta Burst Stimulation protocol (TBS) in slices from 8-month-old WT **(A, C)** and APP/PS1 mice **(B, D)**. A slight decrease in the paired-pulse ratio was observed after TBS in BP- and CP-treated

WT and APP/PS1 slices. However, no significant differences were observed between these 4 groups ($p>0.05$ Kruskal Wallis test).

**Figure S5: BP treatment leads to restoration of Late-LTP in APP/PS1 hippocampal slices. A)** Schematic representation showing the location of the recording electrode (LFP recording) with two stimulating electrodes placed in the stratum radiatum of CA1 to stimulate two independent pathways. Theta burst (TBS) was applied to the simulating electrode (stim1) to evoke LTP in the S1 pathway, whereas the S2 pathway stimulated by the stimulating electrode 2 (stim2) acted as a control. **B)** In the upper panel, pooled data (n = 5) showing the time course of LTP in WT hippocampal slices treated with BP (0.5 μM) 30 to 40 min before TBS induction. TBS applied to the S1 pathway evoked an LTP lasting at least 3 hours (251.8 ± 40.4% from 175 to 180 min, p = 0.019). Below, the time course of synaptic responses is shown for the control pathway (S2) that was not potentiated (107.1 ± 7.8%). The difference in potentiation is significant between the S1 and S2 pathways (p = 0.026). **C)** Pooled data in the upper panel illustrate the time course of LTP in slices derived from APP/PS1 mice (n=5) and treated with the BP (0.5 μM) 30 to 40 min before TBS induction. TBS evoked a long-lasting LTP in S2 pathway up to at least 3 hours (261.8 ± 45.2% from 175 to 180 min, p = 0.023). In contrast, the lower panel show responses from the control pathway (S2) which were not potentiated (98.6 ± 5.2%), demonstrating the specificity of the BP rescue effect on Late-LTP in APP/PS1 slices. Indeed, the difference was significant between the potentiated (S1) and control (S2) pathways (p = 0.015) in APP/PS1 slices, while no difference was observed between potentiated pathways in WT and APP/PS1 slices (p = 0.87). Scale: 200 ms, 0.5 mV.

**Figure S6: Characterization of BP effect on VEGFR2 activation and Aβo-VEGF interaction. A)** Representative immunoblotting for pVEGFR2 and VEGFR2 expression in HUVEC cells treated with PBS (Ctrl), CP or BP for 5 min at 500 nM, in presence or absence of 5 ng.mL$^{-1}$ VEGF. **B)** CP or BP show no effect on basal or VEGF-induced VEGFR2 activation. (Kruskall-Wallis, p = 0,0013, n = 4 ; post-hoc *p<0,05, **p<0,01). **C)** ELISA assay analyzed with a linear model curve fitting showing no impact of CP on Aβo-VEGF interaction. In contrast, a sigmoidal dose response curve fitting shows that BP efficiently prevents Aβo-VEGF interaction in a dose dependent manner. n = 5-6 independent experiments. Error bars represents mean ± SEM.

## TABLE S1 : KEY RESOURCE TABLE

| Reagent or Resource | Source | Identifier |
|---|---|---|
| **Antibodies** | | |
| **Mouse anti β-Amyloid 1-16 monoclonal antibody Dilution 1:500** | Biolegend | Cat#8003002 |
| **Rabbit anti β-Amyloid monoclonal antibody (A11) Dilution 1:1000** | Kindly provided by Rakez Kayed, University of Texas, USA | |
| **Mouse purified anti β-Amyloid 1-16 monoclonal antibody (6E10) Dilution 1:2000** | Biolegend | Cat#803001 |
| **Mouse purified anti β-Amyloid 17-24 monoclonal antibody (4G8) Dilution 1:2000** | Biolegend | Cat#800701 |
| **Rabbit anti β-Amyloid 22-35 polyclonal antibody Dilution 1:500** | MERCK Millipore | Cat#A3356 |
| **Rabbit Anti VEGF Receptor 2 monoclonal antibody. Dilution 1:500** | Cell Signaling Technology | Cat#2479 |
| **Rabbit Anti VEGF Receptor 2 phospho (Tyr1175) monoclonal antibody. Dilution 1:500** | Cell Signaling Technology | Cat#2478 |
| **Goat Anti Rabbit HRP polyclonal antibody Dilution 1:10,000** | Jackson Immunoresearch | Cat# 111-036-003 |
| **Goat Anti Mouse HRP polyclonal antibody Dilution 1:10,000** | Jackson Immunoresearch | Cat# 115-036-003 |
| **Mouse Anti-PSD95 monoclonal antibody Dilution 1:300** | Invitrogen | Cat# 6G6-1C9 |
| **Rabbit Anti-Bassoon polyclonal antibody Dilution 1:500** | Synaptic System | Cat# 141 003 |
| **Goat Anti Rabbit Alexa fluor 555 polyclonal antibody. Dilution 1:1000** | Molecular Probes | Cat# A21429 |
| **Goat Anti Mouse Alexa fluor 488 polyclonal antibody. Dilution 1:1000** | Molecular Probes | Cat# A11029 |
| **Rabbit Anti Biotin Dilution 1:10000** | Abcam | Cat#ab53494 |
| **Chemicals, peptides, and recombinant proteins** | | |
| **Amyloid β protein (1-42)** | Bachem | Cat# H-1368 |
| **Amyloid β protein (1-40)** | Bachem | Cat#4014442 |
| **Biotinyl-Amyloid β protein (1-42)** | Bachem | Cat# H-5642 |
| **Biotinyl-Amyloid β protein (1-40)** | Bachem | Cat# H-5914 |
| **Recombinant Human VEGF 165** | R&D Systems | Cat# 293-VE |
| **Experimental models: cell lines** | | |

| | | |
|---|---|---|
| **HUVEC cells (Human Umbilical Vein Endothelial Cells)** | Promocell | Cat# C12203 |
| **Experimental models: Organisms / Strains** | | |
| ***APP/PS1-21* transgenic mice** | Radde et al., 2006 | N/A |
| **C57BL/6JRj** | Janvier Labs | Cat# SC-C57J-F |
| **Cell culture** | | |
| **Neurobasal medium without phenol red** | ThermoFisher Scientific | Cat#12348017 |
| **B27 supplement** | ThermoFisher Scientific | Cat# 17504044 |
| **Endothelial cell growth medium MV2** | Promocell | Cat#C-22121 |
| **Collagen I** | ThermoFisher Scientific | Cat#A1048301 |
| **Gibco L-Glutamine** | ThermoFisher Scientific | Cat#25030024 |
| **Sodium Pyruvate** | ThermoFisher Scientific | Cat#11530396 |
| **Gibco Penicillin-Streptomycin** | ThermoFisher Scientific | Cat#11548876 |
| **Software and algorithms** | | |
| **Fiji - ImageJ** | MCMaster Biophotonics Facility | Version |
| **Huygens** | Scientific Volume Imaging | Version 16.05 |
| **Icy Bioimage Analysis** | France Bioimaging | Version |
| **R software** | R foundation for Statistical Couputing | R version |
| **Origin Lab** | Origin Lab Corp | Origin Pro Version |
| **Other** | | |
| **Streptavidine Alexa fluor 647** | Invitrogen | Cat# S32357 |
| **Streptavidin HRP** | R&D Systems | Cat#DY998 |
| **Reagent Diluent Concentrate 2** | R&D Systems | Cat#DY995 |
| **Clear polystyrene microplates** | R&D Systems | Cat#DY990 |
| **Stop solution 2N Sulfuric acid** | R&D Systems | Cat#DY994 |
| **Subtrate Reagent Pack** | R&D Systems | Cat#DY999 |
| **Bolt MES SDS Running Buffer** | Thermofisher Scientific | Cat#B0002 |
| **XT Trycin Running Buffer** | Bio-Rad | Cat#1610790 |
| **cOmplete protease inhibitor cocktail** | MERCK Millipore | Cat# 04693159001 |
| **4-12% Criterion XT Bis-Tris Protein Gel** | Bio-Rad | Cat#3450124 |
| **3-8% Criterion XT Tris-Acetate Protein Gel** | Bio-Rad | Cat#3450130 |
| **SuperSignal West Pico Plus Chemiluminescent Subtrate** | ThermoFisher Scientific | Cat#34577 |
| **Sodium orthovanadate** | MERCK Millipore | Cat#S6508 |
| **Phosphatase inhibitor cocktail** | MERCK Milllipore | Cat#524629 |
| **Pierce universal nuclease for cell lysis** | ThermoFisher Scientific | Cat#88701 |

| | | |
|---|---|---|
| **D-PBS no calcium no magnesium** | ThermoFisher Scientific | Cat#D8537 |
| **Thioflavin T** | Sigma-Aldrich | Cat#T3516 |
| **Dapi** | Roche | Cat# 10236276001 |
| **FluorSaveTM reagent** | MERCK Milllipore | Cat# 345789 |

| Figure 1A: ELISA Peptides interaction with Aβ | | | | | | |
|---|---|---|---|---|---|---|
| [Aβ] μg/mL | 0 | 0.01 | 0.1 | 1 | 5 | 10 |
| Aβo/CP | -0.004±0.006 | 0.009±0.007 | 0.028±0.014 | 0.0921±0.102 | 0.074±0.058 | 0.066±0.046 |
| Aβo/BP | 0.000±0.001 | 0.144±0.010 | 0.819±0.115 | 1.262±0.091 | 1.330±0.135 | 1.172±0.108 |
| Aβm/CP | 0.000±0.003 | 0.002±0.002 | 0.009±0.006 | 0.031±0.017 | 0.038±0.026 | 0.012±0.018 |
| Aβm/BP | 0.006±0.003 | 0.049±0.028 | 0.172±0.083 | 0.736±0.376 | 1.034±0.449 | 0.880±0.395 |

| Figure 2: ThT experiment at 24h | | | | | |
|---|---|---|---|---|---|
| [Peptide] μM | 0 μM | 0.5 μM | 1 μM | 5 μM | 15 μM |
| CP | 98.56±1.17 | 93.13±21.58 | 76.68±8.41 | 87.94±9.01 | 69.81±7.05 |
| BP | 98.56±1.17 | 53.49±15.58 | 43.29±16.63 | 17.33±1.23 | 5.02±2.19 |

| Figure 4B-C: Peptides impact on Aβ oligomer formation | | | | | | |
|---|---|---|---|---|---|---|
| Western Blot | 2h | | | 24h | | |
| | Aβ | Aβ+CP | Aβ+BP | Aβ | Aβ+CP | Aβ+BP |
| Monomers % | 10.44±2.5 | 11.08±3.1 | 20.29±4.4 | 8.63±1.1 | 9.54±0.9 | 16.17±1.5 |
| LMW % | 10.46±2.3 | 10.81±1.5 | 20.08±2.4 | 4.72±0.8 | 4.84±0.7 | 12.46±1.9 |
| HMW % | 78.28±4.4 | 77.55±4.5 | 58.95±5.6 | 84.72±0.8 | 83.57±0.8 | 70.85±0.9 |

| Figure 5B: Synaptic targeting | |
|---|---|
| | % Targeted synapses |
| Ctrl+CP | 0.07±0.1 |
| Ctrl+BP | 0.08±0.1 |
| Aβ | 41.1±2.3 |
| Aβ+CP | 40.9±2.3 |
| Aβ+BP | 4.58±3.8 |

| Figure 6G: LTP Experiments | |
|---|---|
| | EPSP slope % |
| WT+CP | 191.4±18.3 |
| WT+BP | 223.8±29.3 |
| APP+CP | 119±9.1 |
| APP+BP | 226.7±40.5 |

| Figure 4D: Peptides impact on Aβ oligomer formation | | | | |
|---|---|---|---|---|
| Dot Blot | 0h | 0.5h | 1h | 2h |
| Aβ % | 100±6.98 | 130.8±9.78 | 161.0±10.6 | 158.7±3.53 |
| Aβ+BP % | 87.4±6.05 | 78.7±8.8 | 73.9±5.1 | 82.4±11.1 |

| Figure S1: Competitive interaction for BP binding to Aβ | | |
|---|---|---|
| | Bound BP | |
| | Monomers | Oligomers |
| No competition | 0.213±0.027 | 0.463±0.069 |
| 6E10 | 0.052±0.015 | 0.089±0.017 |
| 4G8 | 0.086±0.043 | 0.096±0.019 |

| Figure S2B: Synaptic targeting | |
|---|---|
| | % Targeted synapses |
| Ctrl+CP | 0.4±0.2 |
| Ctrl+BP | 0.7±0.3 |
| Aβ | 31.4±2.0 |
| Aβ+CP | 37.5±2.4 |
| Aβ+BP | 34.8±2.3 |

| Figure S3A: Basal synaptic transmission | |
|---|---|
| | EPSP slope % |
| BP 0.5 μM | 102.3 ± 10.50 |
| BP 10 μM | 66.9 ± 9.40 |

| Figure S4: Paired pulse facilitation | |
|---|---|
| | EPSP slope % |
| WT+CP | 96.9 ± 9.60 |
| WT+BP | 88.01 ± 12.80 |
| APP+CP | 93.9 ± 10.20 |
| APP+BP | 94.8 ± 17.50 |

| Figure S5: Late-LTP experiments | |
|---|---|
| | EPSP slope % |
| WT+BP S1 | 251.8±40.4 |
| WT+BP S2 | 107.1±7.8 |
| APP+BP S1 | 261.8 ± 45.2 |
| APP+BP S2 | 98.6±5.2 |

| Figure S6B: Peptide effect on VEGFR2 activation | |
|---|---|
| | VEGFR2 activation |
| Ctrl | 0.055±0.007 |
| Ctrl+CP | 0.085±0.009 |
| Ctrl+BP | 0.154±0.014 |
| VEGF | 1.000±0.090 |
| VEGF+CP | 0.942±0.048 |
| VEGF+BP | 1.167±0.214 |

| Figure S6C: ELISA competition experiment VEGF | | | | | | |
|---|---|---|---|---|---|---|
| [Peptide] μM | 0 | 0.01 | 0.1 | 1 | 10 | 100 |
| CP | 100±0.00 | 92.08±6.40 | 93.80±10.01 | 95.21±10.03 | 99.10±9.07 | 94.55±6.25 |
| BP | 100±0.00 | 76.87±8.37 | 69.45±7.35 | 33.29±15.27 | 1.61±1.80 | 4.03±2.94 |

# TABLE S2

| Figure 2 | |
|---|---|
| Aβ vs Aβ + BP 5μM | Aβ vs Aβ + BP 15μM |
| 0.0483 | 0.0011 |

| | Figure 4B – 2h | | | Figure 4C – 24h | | |
|---|---|---|---|---|---|---|
| | Aβ vs Aβ + CP | Aβ vs Aβ + BP | Aβ + CP vs Aβ + BP | Aβ vs Aβ + CP | Aβ vs Aβ + BP | Aβ + CP vs Aβ + BP |
| monomer | / | / | / | / | 0.0087 | 0.0196 |
| LMW Aβo | / | 0.0243 | 0.0237 | / | 0.0162 | 0.0200 |
| HMW Aβo | / | 0.0294 | 0.0339 | / | 0.0035 | 0.0237 |

| Figure 4E - F | | | | | | |
|---|---|---|---|---|---|---|
| | 0h vs 0.5h | 0h vs 1h | 0h vs 2h | 0.5h vs 1h | 0.5h vs 2h | 1h vs 2h |
| Aβ | / | 0.0055 | 0.0052 | / | / | / |
| Aβ+BP | / | / | / | / | / | / |

| Figure 5B | | | | |
|---|---|---|---|---|
| | Ctrl+BP | Aβ | Aβ+CP | Aβ+BP |
| Ctrl+CP | / | < 0.0001 | < 0.0001 | 0.0488 |
| Ctrl+BP | | < 0.0001 | < 0.0001 | / |
| Aβ+CP | < 0.0001 | / | | 0.0300 |
| Aβ+BP | / | 0.0223 | | / |

| Figure 6 | | |
|---|---|---|
| WT+CP vs APP+CP | WT+BP vs APP+CP | APP+CP vs APP+BP |
| 0.049 | 0.039 | 0.031 |

| Figure S1: Competitive interaction for BP binding to Aβ | | |
|---|---|---|
| | 6E10 | 4G8 |
| No competition monomers | 0.012 | 0.012 |
| 6E10 | | / |
| 4G8 | / | |
| No competition oligomers | 0.012 | 0.012 |
| 6E10 | | / |
| 4G8 | / | |

| Figure S2B : Synaptic targeting | | | | |
|---|---|---|---|---|
| | Ctrl+BP | Aβ | Aβ+CP | Aβ+BP |
| Ctrl+CP | / | 0.01 | 0.0002 | 0.0006 |
| Ctrl+BP | | 0.03 | 0.0012 | 0.003 |
| Aβ+CP | 0.0012 | / | | / |
| Aβ+BP | 0.003 | / | / | |

| Figure S3A: Basal synaptic transmission |
|---|
| BP 0.5 μM vs BP 2 μM |
| 0.014 |

| Figure S5: Late-LTP experiments | |
|---|---|
| WT+BP LTP in S1 (175-180 min) vs WT+BP control in S2 | APP+BP LTP in S1 (175-180 min) vs APP+BP control in S2 |
| 0.026 | 0.015 |

| Figure S6B: Peptide effect on VEGFR2 activation | | |
|---|---|---|
| Ctrl vs VEGF | Ctrl vs VEGF+CP | Ctrl vs VEGF+BP |
| 0.0114 | 0.0106 | 0.007 |

# TABLE S3
Statistics

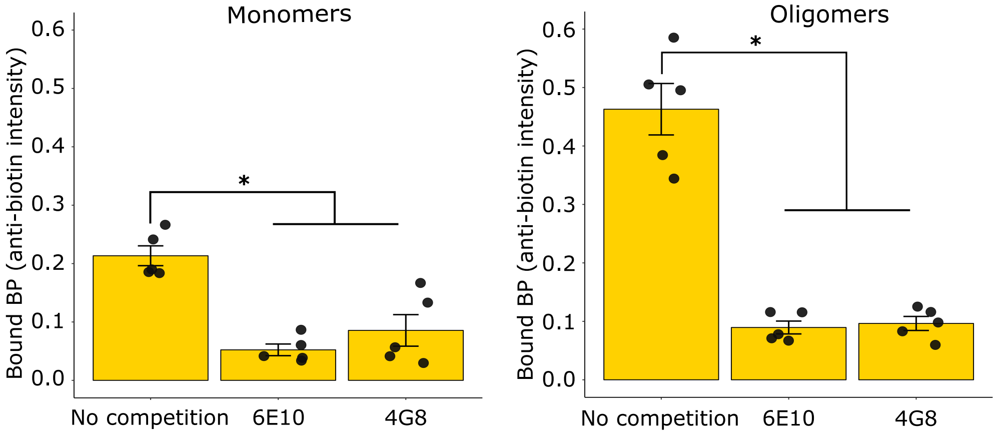
Monomers
Oligomers
Bound BP (anti-biotin intensity)
0.6
0.5
0.4
0.3
0.2
0.1
0.0
*
No competition
6E10
4G8

Figure S1

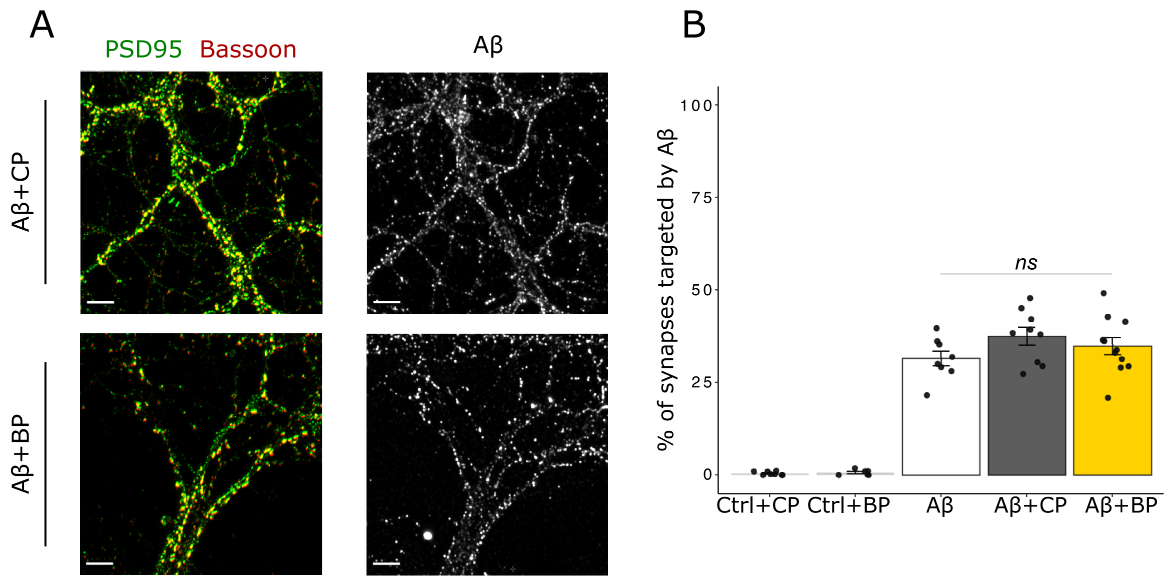
A
PSD95 Bassoon
Aβ
Aβ+CP
Aβ+BP
B
% of synapses targeted by Aβ
100
75
50
25
0
ns
Ctrl+CP
Ctrl+BP
Aβ
Aβ+CP
Aβ+BP

Figure S2

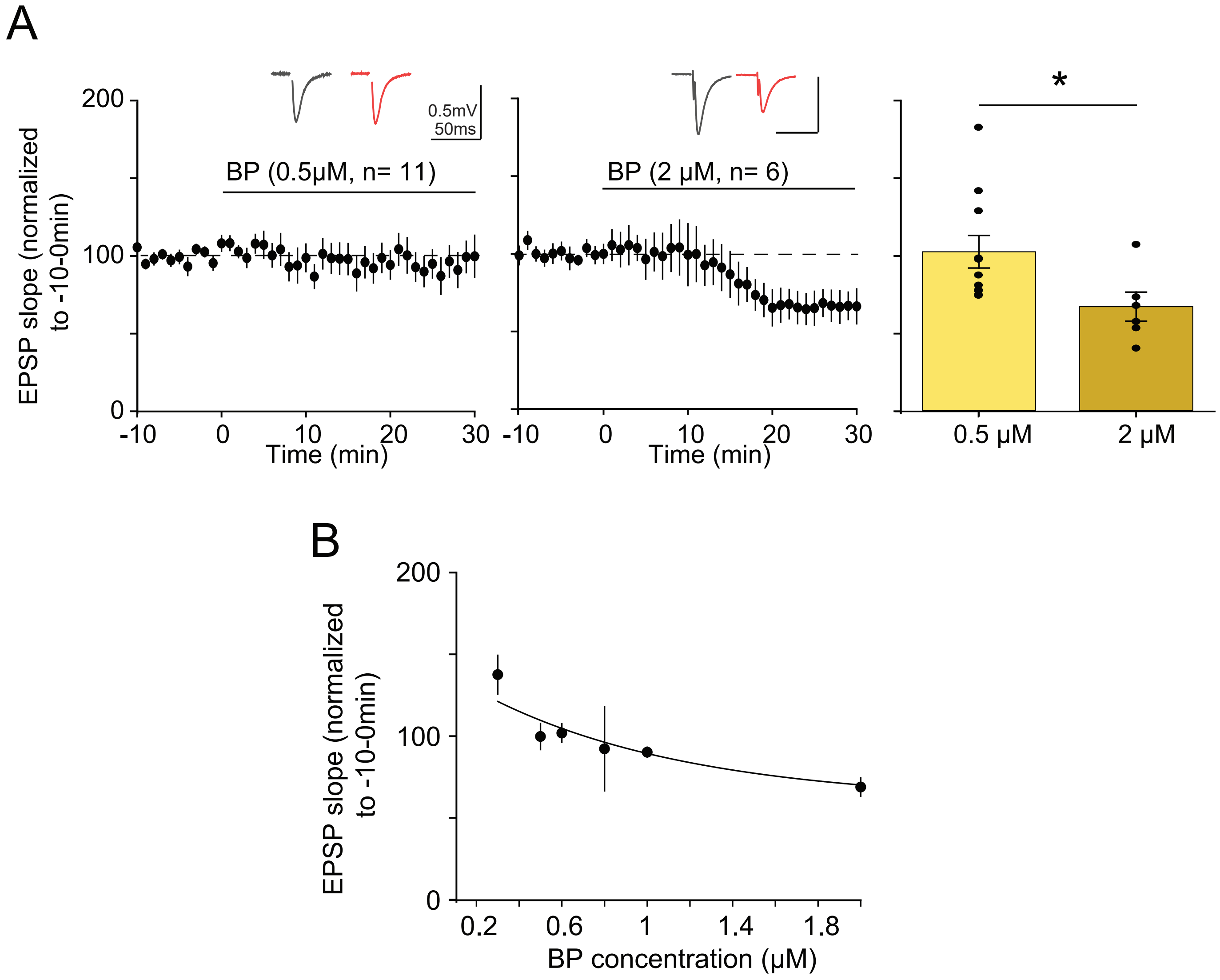
A
EPSP slope (normalized to -10-0min)
200
100
0
-10
0
10
20
30
Time (min)
BP (0.5μM, n= 11)
0.5mV
50ms
BP (2 μM, n= 6)
*
0.5 μM
2 μM
B
BP concentration (μM)
0.2
0.6
1
1.4
1.8

Figure S3

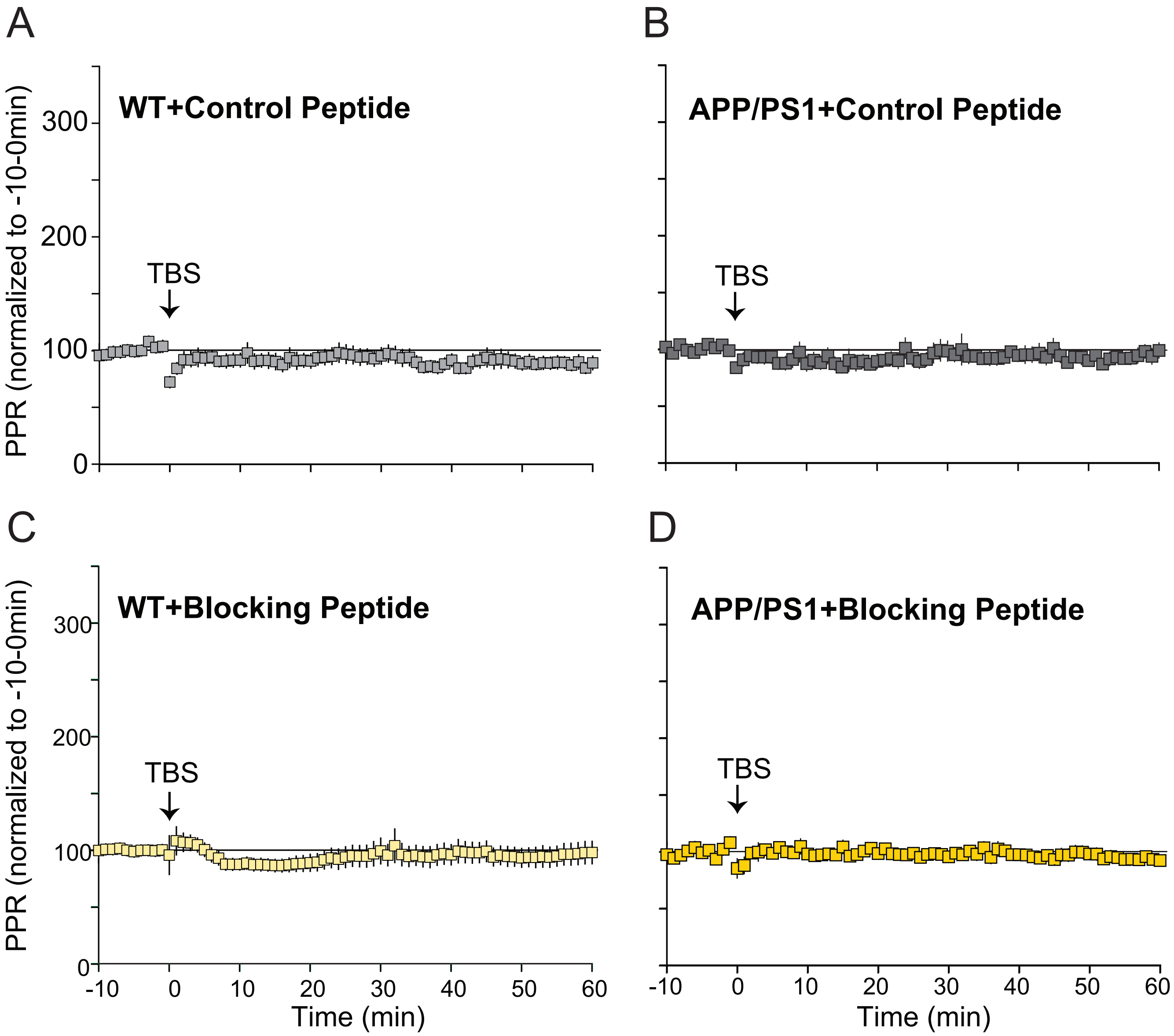
A
B
C
D
WT+Control Peptide
APP/PS1+Control Peptide
WT+Blocking Peptide
APP/PS1+Blocking Peptide
TBS
PPR (normalized to -10-0min)
Time (min)
0
100
200
300
-10
0
10
20
30
40
50
60

Figure S4

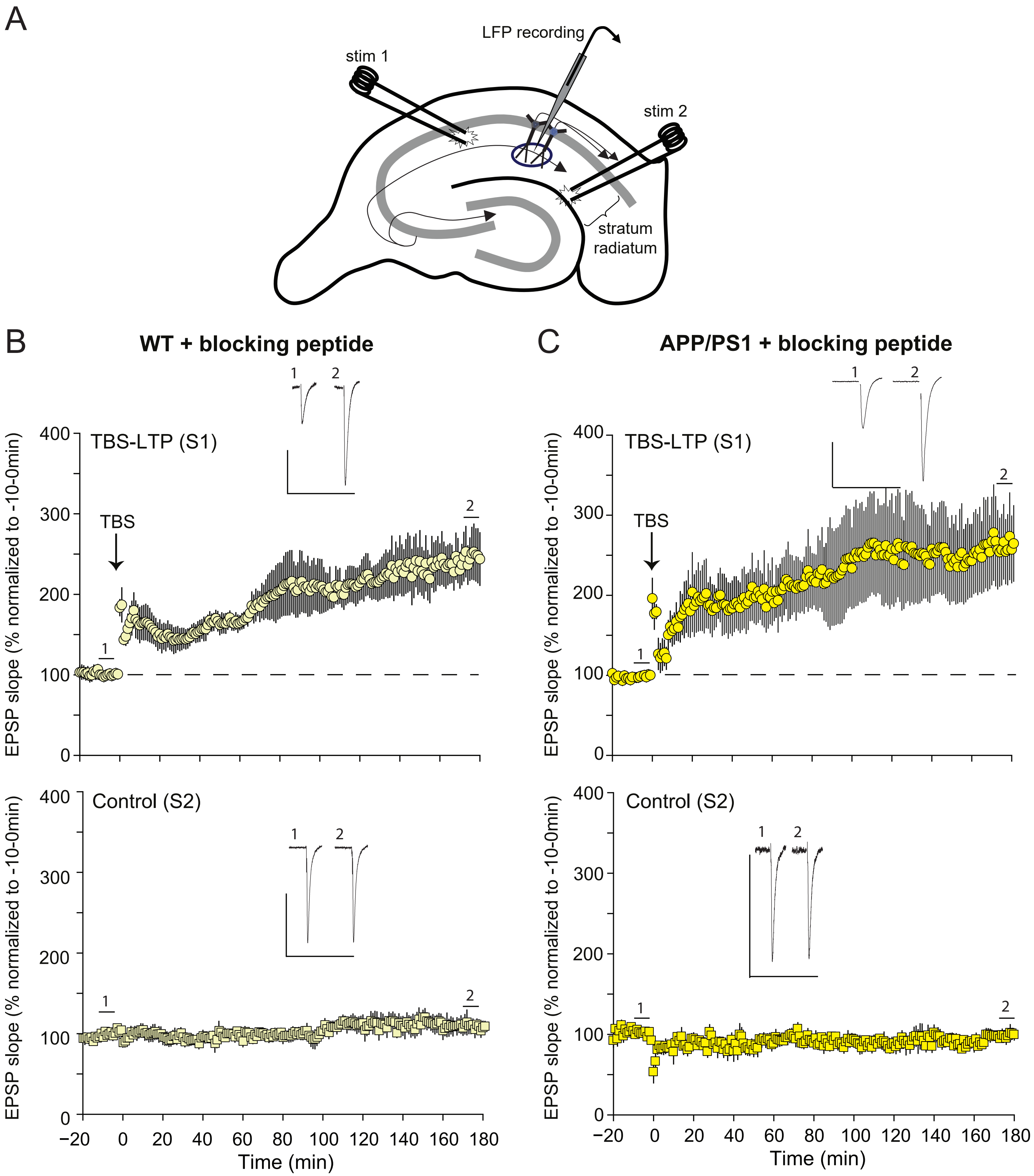
A
LFP recording
stim 1
stim 2
stratum
radiatum
B
WT + blocking peptide
C
APP/PS1 + blocking peptide
TBS-LTP (S1)
TBS
Control (S2)
EPSP slope (% normalized to -10-0min)
Time (min)

Figure S5

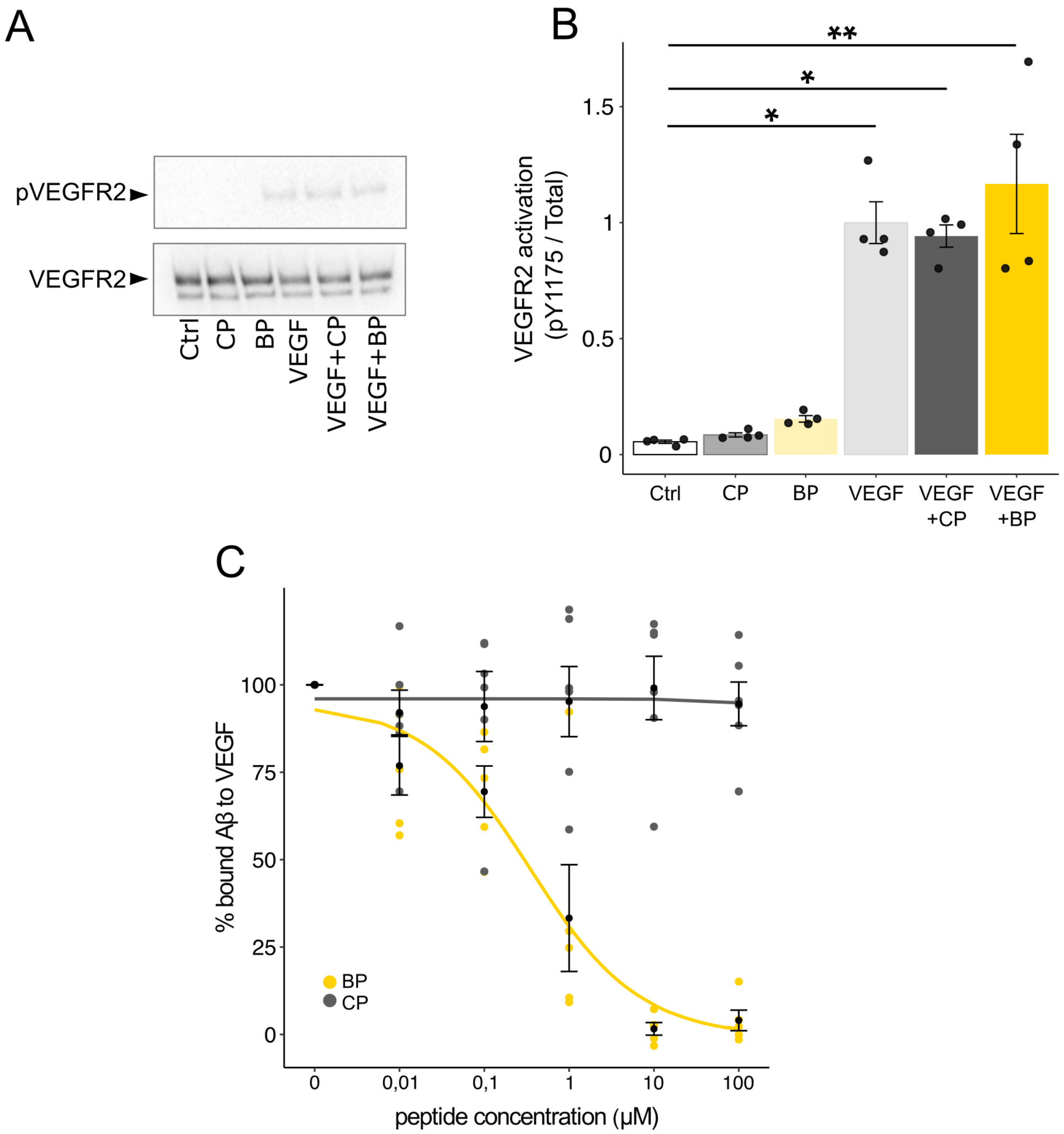
A
pVEGFR2
VEGFR2
Ctrl
CP
BP
VEGF
VEGF+CP
VEGF+BP
B
VEGFR2 activation
(pY1175 / Total)
**
*
*
1.5
1
0.5
0
Ctrl
CP
BP
VEGF
VEGF +CP
VEGF +BP
C
% bound Aβ to VEGF
100
75
50
25
0
BP
CP
0
0,01
0,1
1
10
100
peptide concentration (μM)

Figure S6
