# Supplementary material for: A novel peptide derived from vascular endothelial growth factor prevents amyloid beta aggregation and toxicity
Source: Aging Cell. 2023 Jul 6;22(9):e13907. doi: 10.1111/acel.13907 (PMC10497828; doi:10.1111/acel.13907)
Supplement: Supplementary file 2 — Appendix S2 [file ACEL-22-e13907-s002.pdf]

SUPPORTING TABLE LEGENDS

Table S1: Key resources table

Summary of the wild type and genetically modified mice strains, cell line, antibodies, recombinant proteins and synthetic peptides, cell culture media and reagents, as well as software and algorithms used in the present study.

Table S2: Summary of the data related to Figures 2, 4, 5, 6, S1, S2, S3, S5 and S6

Data are presented as means ± SEM. They refer to ELISA absorbance values reflecting CP or BP binding as a function of Aβm or Aβo concentration, as shown in Figure 1. Normalized ThT fluorescence data are indicated as a function of CP or BP concentration, as illustrated in Figure 2. Percentages of various Aβ species present after 2h or 24h of Aβ aggregation in presence of CP or BP are listed for western blotting experiments, as shown in of Figure 4. Percentages are also stated for dot blot experiments of the same Figure. Percentages of full hippocampal synapses positive for Aβ immunostaining in CP or BP treated cultures are listed, as illustrated in Figure 5 and Figure S2. Normalized fEPSP slopes obtained after TBS in CP or BP-treated hippocampal slices derived from WT or APP/PS1 mice are indicated, as shown in Figure 6 and Figure S5. In addition, normalized fEPSP slopes measured at baseline and after TBS are used to compare the paired-pulse ratio (PPR) overtime, as illustrated in Figure S4. Optical density values illustrating immunoreactivity in the presence or absence of 6E10 or 4G8 competition are reported, as quantified in Figure S1. Normalized fEPSP slopes obtained after various concentrations of BP treatment in WT hippocampal slices are reported, as shown in Figure S3. Finally, referring to Figure S6, optical density ratios reflecting VEGFR2 activation level are indicated, as well as normalized ELISA absorbance values measuring Aβo binding to VEGF in the presence of CP or BP.

Table S3: Summary showing statistical analyses of the data reported in Table S2

Results of post-hoc comparisons showing significant differences are indicated with Holm correction for multiple comparisons.
